# Design optimization of a magnesium-based metal hydride hydrogen energy storage system

**DOI:** 10.1038/s41598-022-17120-3

**Published:** 2022-08-04

**Authors:** Puchanee Larpruenrudee, Nick S. Bennett, YuanTong Gu, Robert Fitch, Mohammad S. Islam

**Affiliations:** 1 School of Mechanical and Mechatronic Engineering, University of Technology Sydney (UTS), 15 Broadway, Ultimo, NSW 2007 Australia; 2 School of Mechanical, Medical and Process Engineering, Faculty of Engineering, Queensland University of Technology, Brisbane, 4000 Australia

**Keywords:** Engineering, Mechanical engineering

## Abstract

Metal hydrides (MH) are known as one of the most suitable material groups for hydrogen energy storage because of their large hydrogen storage capacity, low operating pressure, and high safety. However, their slow hydrogen absorption kinetics significantly decreases storage performance. Faster heat removal from MH storage can play an essential role to enhance its hydrogen absorption rate, resulting in better storage performance. In this regard, the present study aims to improve heat transfer performance to positively impact the hydrogen absorption rate of MH storage systems. A novel semi-cylindrical coil is first designed and optimized for hydrogen storage and embedded as an internal heat exchanger with air as the heat transfer fluid (HTF). The effect of novel heat exchanger configurations is analyzed and compared with normal helical coil geometry, based on various pitch sizes. Furthermore, the operating parameters of MH storage and HTF are numerically investigated to obtain optimal values. ANSYS Fluent 2020 R2 is utilized for the numerical simulations. Results from this study demonstrate that MH storage performance is significantly improved by using a semi-cylindrical coil heat exchanger (SCHE). The hydrogen absorption duration reduces by 59% compared to a normal helical coil heat exchanger. The lowest coil pitch from SCHE leads to a 61% reduction of the absorption time. In terms of operating parameters for the MH storage with SCHE, all selected parameters provide a major improvement in the hydrogen absorption process, especially the inlet temperature of the HTF.

## Introduction

A move away from fossil fuel-based energy resources towards renewable forms of energy is underway at a global scale. Since many forms of renewable energy provide electricity in a dynamic way, energy storage is required to balance load. Hydrogen-based energy storage is receiving much attention for this purpose, not least because hydrogen can be employed as a ‘green’ alternative fuel and energy storage medium, because of its characteristics and portability^1^. Furthermore, hydrogen also offers a higher energy capacity per mass compared to fossil fuels^2^. There are four main types of hydrogen energy storage: compressed gas, underground storage, liquid storage, and solid storage. Compressed hydrogen gas is the main type that has been used in fuel-cell vehicles such as buses and forklifts. However, this storage provides a low volumetric hydrogen density (around 0.089 kg/m^3^) and presents safety concerns regarding high operating pressure^3^. Liquid storage will store hydrogen in liquid form, based on the converting process with a low temperature and ambient pressure. However, there is around 40% energy loss during the liquefaction process. Moreover, this technique is also known for higher energy consumption as well as being time-consuming compared to the solid storage technique^4^. Solid storage is a feasible option for the hydrogen economy that stores hydrogen by combining it within solid materials through absorption and releasing hydrogen through desorption^5^. Metal hydride (MH) is one of the solid material storage technologies that has recently attracted significant interest in fuel cell applications because of having a high hydrogen capacity, low operating pressure, and low cost compared to liquid storage, for both stationary and mobile applications^6,7^. Moreover, MH materials also offer safe performance as high-volume efficiency storage^8^. However, there is one problem that limits MH performance: MH reactors suffer from low thermal conductivity^9^, resulting in slow hydrogen absorption and desorption.

Appropriately transferring heat during the exothermic and endothermic reactions is the key to improving MH reactor performance. For the hydrogen charging process, the generated heat must be removed from the reactor in order to control the hydrogen charging flow at the desired rate with the maximum storage capacity^10^. In contrast, heat is required to improve the hydrogen release rate during the discharging process. To improve the heat and mass transfer performances, many researchers have studied the design and optimization based on several factors including operating parameters, MH structure, and MH optimization^11^. MH optimization can be done by adding high thermal conductivity materials such as the metal foams into the MH bed^12,13^. By this method, the effective thermal conductivity can be increased from 0.1 up to 2 W/mK^10^. However, adding solid material significantly reduces the MH reactor capacity. For the operating parameters, improvements can be achieved by optimization of the initial operating conditions of the MH bed and heat transfer fluid (HTF). The MH structure can be optimized by the reactor’s geometry and the arrangement of heat exchanger designs^14^. In terms of heat exchanger configuration of the MH reactor, approaches can be classified into two types. These are an internal heat exchanger, which is embedded in the MH bed, and an external heat exchanger such as fins, cooling jacket and water bath that cover the MH bed^15^. For external heat exchanger, Kaplan^16^, analyzed the performance of a MH reactor by employing cooling water as a jacket to reduce the temperature inside the reactor. The results were compared to a reactor with 22 circular fins and another reactor that cools by natural convection. They claimed that having a cooling jacket significantly reduced MH temperature resulting in a better absorption rate. The numerical study of the MH reactor with water jacket from Patil and Gopal^17^, indicated that the hydrogen supply pressure and temperature of HTF are the key parameters to affect the hydrogen absorption and desorption rates.

Increasing heat transfer area by adding fins and heat exchangers embedded inside MHs are key for improving heat and mass transfer characteristics that lead to the enhancement of MH storage performance^18^. Several internal heat exchanger configurations (straight tube and helical coil tube) have been developed in order to circulate cooling fluid throughout the MH reactor^19–26^. With an internal heat exchanger, the cooling or heating fluid will transfer local heat inside the MH reactor during the hydrogen sorption processes. Raju and Kumar^27^, employed several straight tubes as heat exchangers to improve MH performance. Their results indicated that the absorption time was reduced when using straight tubes as heat exchangers. Similarly, using a straight tube also reduced the hydrogen desorption time^28^. A higher flow rate of cooling fluid increases hydrogen charging and discharging rates^29^. However, increasing the number of cooling tubes positively affects MH performance rather than the flow rate of cooling fluid^30,31^. Raju et al.^32^, investigated the performance of multi-tube heat exchangers inside the reactor by using LaMi_4.7_Al_0.3_ as MH materials. They reported that the operating parameters significantly affect the absorption process, especially supply pressure, followed by HTF flow rate. However, the absorption temperature was found to be less significant.

The performance of MH reactors was further improved by utilizing a helical coil heat exchanger, as it enhances the heat transfer compared to straight tubes. This is because of secondary circulations that result in better heat removal from the reactor^25^. Moreover, the helical tube provides more surface area for heat removal from the MH bed to the cooling fluid. This method also produces a more uniform distribution of the heat transfer tubes when it is embedded inside the reactor^33^. Wang et al.^34^, studied the effect of hydrogen absorption duration by adding a helical coil in the MH reactor. Their results indicated that the absorption time decreased when increasing the heat transfer coefficient of the heat transfer fluid. Wu et al.^25^, studied the performance of a MH reactor based on Mg_2_Ni and helical coil heat exchanger. Their numerical study showed a reduction in the reaction time. The enhancement of the heat transfer mechanism in a MH reactor is based on a smaller ratio of helical pitch to the helical diameter and non-dimensional pitch. The experimental study of using a helical coil as an internal heat exchanger by Mellouli et al.^21^ proved that the initial temperature of HTF significantly affects the improvement of hydrogen absorption and desorption times. The combination of various internal heat exchangers has been made by several studies. Eisapour et al.^35^, studied MH storage by employing a helical coil heat exchanger along with a central return tube in order to improve the hydrogen absorption process. Their results indicated that a helical tube along with a central return tube significantly improved heat exchanged between cooling fluid and MH. A lower pitch of the helical tube and a higher tube diameter increased the heat and mass transfer rate. Ardahaie et al.^36^, employed flat spiral tube planes as a heat exchanger for heat transfer enhancement inside a reactor. They reported that the absorption duration was reduced by increasing the number of flat spiral tube planes. The combination of various internal heat exchangers has been made by several studies. Dhaou et al.^37^, improved the MH performance by employing both helical coil heat exchanger and fins. Their results showed that this technique reduces hydrogen refilling time which is a 2 times reduction comparing to without fins. The annular fin was incorporated with the cooling tube and embedded inside the MH reactor^38^. The results from this study showed that this combination technique obtains more uniform heat transfer compared to the MH reactor without using fin. However, combining various heat exchangers will negatively affect the gravimetric and volumetric of the MH reactor. A comparison of different heat exchanger configurations was made by Wu et al.^18^. These included a straight tube, fins, and helical coil. The authors reported that the helical coil has the best effects on the heat and mass transfer improvements. Similarly, a double coiled tube has a better effect on the heat transfer enhancement compared to a straight tube, spiral tube, and straight tube incorporating with spiral tube^39^. The study from Sekhar et al.^40^, proved that using a helical coil as an internal heat exchanger and an external cooling jacket with fins obtained a similar improvement in hydrogen absorption.

From the above mentioned example, using a helical coil as an internal heat exchanger offers a better heat and mass transfer improvement compared to other heat exchangers, especially straight tube and fin. Therefore, the aim of this study is to further develop a helical coil to increase heat transfer performance. A novel semi-cylindrical coil has been firstly developed from the traditional helical coil for MH storage. The expectation from this study is to enhance hydrogen storage performance due to the structure of a novel heat exchanger that provides a better heat transfer area arrangement by considering the constant volume of the MH bed and HTF tube. The storage performance of this novel heat exchanger is then compared with a normal helical coil heat exchanger based on various coil pitches. From the available literature, the operating conditions and coil pitch are the main factors that affect the MH reactor’s performance. To optimize the design of this novel heat exchanger, the effect of coil pitch on the hydrogen absorption time and the MH volume is investigated. Furthermore, to understand the relationship between a novel semi-cylindrical coil and the operating conditions, the secondary aims of this present study are to investigate the reactor’s performance based on various operating parameter ranges and identify an appropriate value for each operating parameter.

## System description

The performance of hydrogen energy storage in this study is investigated based on two heat exchanger configurations (including a helical tube for case 1 to case 3 and a semi-cylindrical tube for case 4 to case 6), and sensitivity analysis on the operating parameters. The performance of a MH reactor is firstly examined based on the helical tube as a heat exchanger. Both HTF tube and the outer shell of the MH reactor are made of stainless steel. It should be noted that the size of MH reactor and the diameter of the HTF tube is constant for all cases, while the HTF pitch sizes vary. In this section, the impact of HTF coil pitch sizes is analyzed. The height and outer diameter of the reactor are 110 mm and 156 mm, respectively. The diameter of HTF tube is fixed as 6 mm. The detail regarding a schematic diagram of MH reactors with a helical tube and two semi-cylindrical tubes can be found in the Supplementary section.

### Metal hydride reactor with helical coil heat exchanger and semi-cylindrical coil heat exchanger

**Figure 1:**
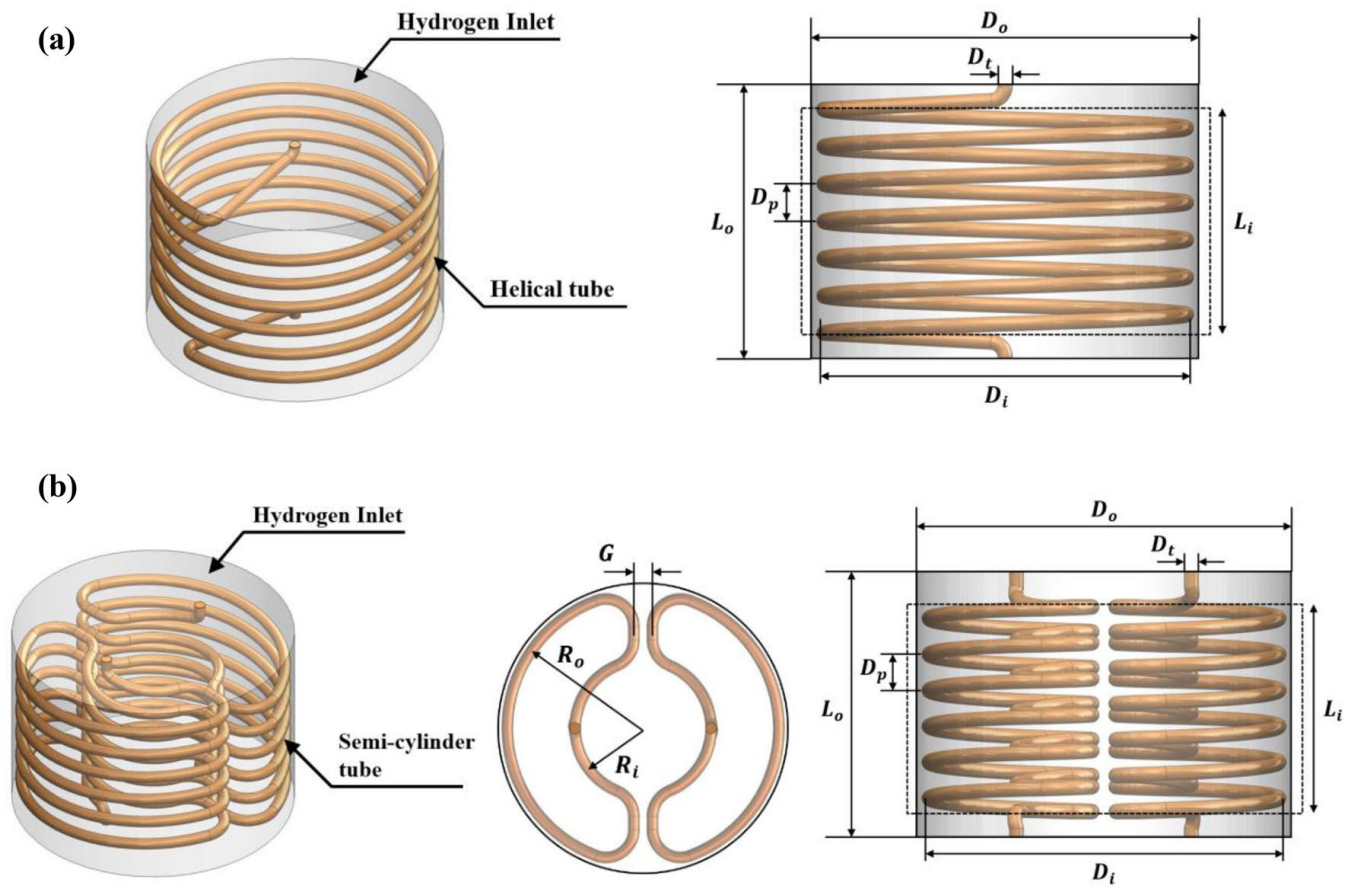
Characteristics of selected geometries for metal hydride reactors. (**a**) With helical tube heat exchanger, and (**b**) with semi-cylindrical tube heat exchanger.

Figure 1a presents the MH reactors with a helical tube and its dimensions. All geometrical parameters are provided in Table 1. The total helical tube volume and MH volume are approximately 100 cm^3^ and 2000 cm^3^, respectively. From this MH reactor, air as the HTF is injected from the bottom part into the porous MH reactor through a helical tube, while hydrogen is injected from the upper surface of the reactor.

**Table 1:** Geometrical characteristics of MH reactors for the helical tube (case 1 to case 3) and the semi-cylindrical tube (case 4 to case 6).

Case No.	${D}_{i}$ (mm)	${D}_{o}$ (mm)	${D}_{p}$ (mm)	${D}_{t}$ (mm)	$G$ (mm)	${L}_{i}$ (mm)	${L}_{o}$ (mm)	${R}_{o}$ (mm)	${R}_{i}$ (mm)
Case 1	Equal: 146	Equal: 156	15	Equal: 6	–	Equal: 90	Equal: 110	–	–
Case 2			12.86		–			–	–
Case 3			10		–			–	–
Case 4			15		Equal: 10			Equal: 73	Equal: 36.5
Case 5			12.86						
Case 6			10						

In the second part, the performance of the MH reactor is then investigated based on the semi-cylindrical tubes as heat exchangers. Figure 1b shows the MH reactors with two semi-cylindrical tubes and their dimensions. Table 1 presents all geometrical parameters for a semi-cylindrical tube that are kept constant except the pitch sizes. It should be noted that the semi-cylindrical tube from case 4 was designed by considering the constant volume of HTF tube and MH alloys from the helical tube (case 3). Regarding Fig. 1b, air is also injected from the bottom part for both HTF semi-cylindrical tubes, whereas hydrogen is injected from the opposite direction of MH reactor.

### Sensitivity analysis

Due to the new design of the heat exchanger, the objective of this section is to identify appropriate initial values for the operating parameters of the MH reactor that is incorporated with SCHE. For all cases, air is employed as the HTF to remove the heat from the reactor. Among HTFs, air and water are commonly selected as the HTF for the MH reactor due to having a low cost and less environmental impact. Due to a high operating temperature range of magnesium-based alloy, air is selected as the HTF for the present study. Moreover, it also has better flow characteristics compared to other liquid metals and molten salt^41^. Table 2 represents the properties of air at 573 K. For sensitivity analysis, only the best configuration of MH-SCHE performance case (among case 4 to case 6) is then applied to this section. This section is evaluated based on various operating parameters, including an initial temperature of the MH reactor, loading pressure of hydrogen, inlet temperature of HTF, and the Reynolds number, which is calculated by changing the velocity of the HTF. All operating parameters for sensitivity analysis are included in Table 3.

**Table 2:** Thermo-physical properties of air at 573 K^35^.

Parameters	Symbols	Values
Density	${\rho }_{f}$	0.60875 kg m^−3^
Specific heat	${C}_{p,f}$	1045 J kg^−1^ K^−1^
Thermal conductivity	${\uplambda }_{f}$	0.045 W m^−1^ K^−1^
Dynamic viscosity	${\mu }_{f}$	2.061 × 10^–5^ Pa s

**Table 3:** Operating conditions for sensitivity analysis.

Operating parameters	Base value	Sensitivity values
Initial temperature of MH reactor (K)	573	473, 523, 623
Loading pressure of hydrogen (MPa)	1.8	1.2, 2.4, 3.0
Inlet temperature of HTF (K)	573	373, 473, 673
Reynolds number of HTF	14,000	10,000, 18,000, 22,000

## Mathematical model

This section describes all the necessary governing equations for the hydrogen absorption process, turbulent flow and heat transfer of the heat transfer fluid.

To simplify the solution of the hydrogen absorption reaction, the following assumptions are made and provided;

- During the absorption process, the thermo-physical properties of hydrogen and metal hydride are constant^40^.
- The radiation heat transfer is neglected in the metal hydride reactor^42^.
- Hydrogen is considered as an ideal gas, local thermal equilibrium conditions are therefore considered^43,44^.
- The pressure gradient effect of hydrogen injection is negligible^45^.
  1 $$N=\frac{{\uplambda }_{e}M{L}_{gas}^{2}\mu }{{P}_{aeq}\frac{{\Delta H}^{2}}{{RT}^{2}}{\rho }_{g}K{L}_{heat}^{2}}$$
  where ${L}_{gas}$ is the tank radius and ${L}_{heat}$ is the axial height of the tank. The hydrogen flow in the tank can be omitted in the simulation without obtaining a significant error when *N* is less than 0.01^46^. From this present study, *N* is far lower than 0.1. Therefore, the pressure gradients effect is negligible.
- The reactor’s walls for all cases are well insulated. Thus, there is no heat transfer between the reactor and the ambient^47^.

Magnesium-based alloys are known for having favourable hydrogeneration properties as well as a high hydrogen storage capacity, that is up to 7.6 wt%^8^. In terms of solid-state hydrogen storage applications, these alloys are also known as lightweight materials. Moreover, they also have excellent heat resistivity and good recyclability^8^. Among several magnesium-based alloys, magnesium-nickel alloys based on Mg_2_Ni is one of the most suitable choices for MH storage due to the hydrogen storage capacity that can be up to 6 wt%. Mg_2_Ni alloys also provide faster kinetics of absorption and desorption processes compared to magnesium hydride^48^. Therefore, Mg_2_Ni is selected in this study as the metal hydride material.

### Governing equations

#### Absorption process

The energy equation is expressed based on the thermal equilibrium between hydrogen and Mg_2_Ni hydride as^25^:

2 $$\frac{\partial \left({\left(\rho {C}_{p}\right)}_{e,MH}T\right)}{\partial t}=\nabla \cdot \left({\uplambda }_{e,MH}\nabla T\right)+\frac{{\rho }_{MH}wt\left(1-\varepsilon \right)\Delta H}{{M}_{{H}_{2}}}\frac{dX}{dt},$$

where the effective heat capacity and conductivity are given as:

3 $${\left(\rho {C}_{p}\right)}_{e,MH}={\varepsilon }_{MH}\times {\rho }_{{H}_{2}}\times {C}_{p,{H}_{2}}+\left(1-{\varepsilon }_{MH}\right)\times {\rho }_{MH}\times {C}_{p,MH}$$

4 $${\uplambda }_{e,MH}= {\varepsilon }_{MH}\times {\uplambda }_{H2}+\left(1-{\varepsilon }_{MH}\right)\times {\uplambda }_{MH}.$$

The hydrogenation reaction of Mg_2_Ni bed ($\Delta H$) is determined as:

5 $${Mg}_{2}Ni+{2H}_{2}\leftrightarrow {Mg}_{2}Ni{H}_{4}+\Delta H.$$

X is the amount of hydrogen absorption on the metal surface in $wt\%$ that is calculated from the kinetic equation in the absorption process $\frac{dX}{dt}$ as follow^49^:

6 $$\frac{dX}{dt}={C}_{a}\left(\frac{{P}_{{H}_{2}}-{P}_{a,eq}}{{P}_{a,eq}}\right)\left(\frac{x-{x}_{f}}{{x}_{0}-{x}_{f}}\right){exp}^{\left(\frac{-{E}_{a}}{RT}\right)},$$

where ${C}_{a}$ denotes the reaction rate and ${E}_{a}$ refers to the activation energy. ${P}_{a,eq}$ is the equilibrium pressure inside the metal hydride reactor for the absorption process which is determined using the Van’t Hoff equation as follows^25^:

7 $$\frac{{P}_{a,eq}}{{P}_{ref}}={exp}^{\left(\frac{\Delta H}{R{T}_{m}}-\frac{\Delta S}{R}\right)},$$

where ${P}_{ref}$ is the reference pressure of 0.1 MPa. $\Delta H$ and $\Delta S$ are the reaction enthalpy and reaction entropy, respectively. The properties of Mg_2_Ni alloys and hydrogen are provided in Table 4. A list of nomenclatures can be found in the Supplementary section.

**Table 4:** Thermo-physical properties of hydrogen and metal hydride in model equations^25,51^.

Parameters	Symbols	Values
Initial temperature	${T}_{0}$	573 K
Inlet temperature of HTF	${T}_{i,HTF}$	573 K
Hydrogen exerting pressure	${P}_{0,H2}$	1.8 MPa
Molecular weight of MH	${M}_{MH}$	0.1073 kg mol^−1^
Hydride specific heat	${C}_{p,MH}$	1414 J kg^−1^ K^−1^
Density of MH	${\rho }_{MH}$	3200 kg m^−3^
Density of saturated MH	${\rho }_{ss,MH}$	3319.32 kg m^−3^
Reaction enthalpy	$\Delta H$	− 6336 J mol^−1^
Reaction entropy	$\Delta S$	− 120.84 J mol^−1^ K^−1^
Reaction rate constant	${C}_{a}$	175.07 s^−1^
Activation energy	${E}_{a}$	49,674 J mol^−1^
Porosity	$\varepsilon$	0.5
Effective thermal conductivity of MH	${\uplambda }_{MH}$	0.674 W m^−1^ K^−1^
Maximum concentration of hydrogen in the MH	${x}_{f}$	1.0
Initial concentration of hydrogen in the MH	${x}_{0}$	0.043
Maximum mass content of hydrogen in the metal	$wt$	3.6%
Permeability	$K$	1 × 10^–8^ m^2^
Density of hydrogen	${\rho }_{H2}$	0.32 kg m^−3^
Thermal conductivity of hydrogen	${\uplambda }_{H2}$	0.167 W m^−1^ K^−1^
Specific heat of hydrogen	${C}_{p,H2}$	14,890 J kg^−1^ K^−1^
Dynamic viscosity of hydrogen	${\mu }_{H2}$	8.41 × 10^–6^ Pa s
Molecular weight of hydrogen	${M}_{H2}$	0.002 kg mol^−1^

#### Heat transfer fluid

The fluid flow is considered as turbulent due to its velocity and the Reynolds number (Re), which are 78.75 m s^−1^ and 14,000, respectively. The realizable k–ε turbulence model is selected in this present study. It was observed that this method provides more accuracy when compared with other k–ε methods and also provides less computational time than the RNG k–ε method^50,51^. Details about the governing equation for heat transfer fluid can be found in the Supplementary section.

### Initial and boundary conditions

At the initial time, uniform conditions are applied for the temperature inside the MH reactor with the average concentration of hydrogen as 0.043. The outer boundary of the MH reactor is assumed to be well insulated. The magnesium-based alloys usually require a high operating temperature for the reaction to store and release the hydrogen from the reactor. For the Mg_2_Ni, this alloy requires the temperature range of 523–603 K to achieve the maximum absorption and the temperature range of 573–603 K to complete the desorption^52^. However, the experimental study by Muthukumar et al.^53^ proved that using the operating temperature at 573 K could achieve the maximum hydrogen storage capacity of the Mg_2_Ni storage which is equal to its theoretical capacity. Therefore, the temperature at 573 K is selected for the initial temperature of the MH reactor in the present study.

8 $${T}_{MH}={T}_{0}=573\, {\mathrm{K}},\,\,\,{P}_{0}=1.8\, {\mathrm{MPa}},\,\,\,{x}_{0}=0.043$$

1. At the shell of the reactor:
  9 $$\frac{\partial {T}_{MH}}{\partial \overrightarrow{n}}=0,$$
2. At the heat transfer fluid inlet
  10 $${u}_{x}={u}_{z}=0,\,\,\,{u}_{y}={u}_{in},\,\,\,T={T}_{in},$$
3. At the heat transfer fluid outlet
  11 $$p={p}_{s}={p}_{a}.$$

### Grid independency

**Figure 2:**
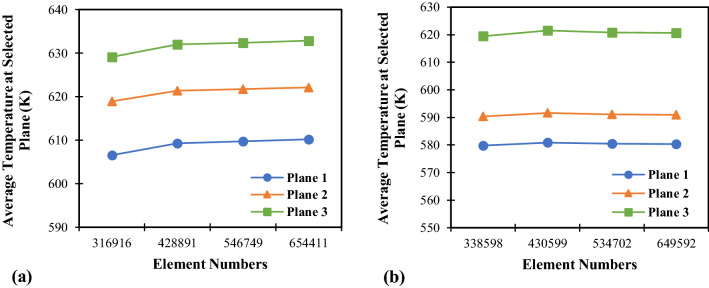
Average bed temperature at selected location for the hydrogen absorption process in the metal hydride reactor under various grid numbers. (**a**) Average temperature at selection location for case 1, and (**b**) average temperature at selected location for case 4.

Various grid sizes are established in order to verify and achieve reliable results. The average temperature at selected locations for the hydrogen absorption process from four different element numbers are provide in Fig. 2. It is worth mentioning that only one case for each configuration is selected for grid independency checks due to having similar geometries. The same meshing methods are applied to other cases. Therefore, case 1 for the helical tube and case 4 for the semi-cylindrical tube are chosen. Figure 2a,b demonstrates the average temperature in the reactor from case 1 and case 4, respectively. The three selected locations represent the bed temperature contour at the top, middle, and bottom parts of the reactor. From temperature contours at the selected locations, the average temperature becomes stable and shows minor changes at the element numbers of 428,891 and 430,599 for case 1 and case 4, respectively. Therefore, these grid sizes are selected for further computational calculations. The details about the average bed temperature for the hydrogen absorption process for various mesh size and the successively refined grids for these two cases can be found in the Supplementary section.

### Model validation and numerical schemes

#### Validation for metal hydride bed and turbulence model

**Figure 3:**
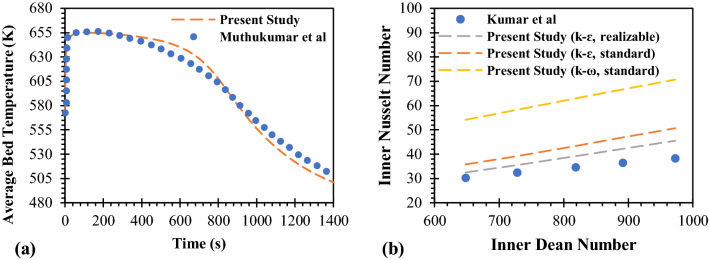
Model validation. (**a**) Code validation of the Mg_2_Ni metal hydride reactor by the comparison of present study and experimental works from Muthukumar et al.^52^, and (**b**) validation study of the turbulence model in helical tube by the comparison of present study and Kumar et al.^54^.

The magnesium-based metal hydride reactor from this present study is validated against experimental results from Muthukumar et al.^53^. In their study, they employed Mg_2_Ni alloy for hydrogen storage with a stainless-steel tube. The copper fins were used to improve the heat transfer inside the reactor. Figure 3a shows the comparison of the average bed temperature for the absorption process between the experimental study and the present study. The selected operating conditions from this experiment are 573 K for the initial MH temperature and 2 MPa for supply pressure. According to Fig. 3a, it is clearly shown that there is a good agreement between this experimental and present results in terms of the average bed temperature.

To validate the turbulence model, the results from this present study are compared with the experimental results from Kumar et al.^54^, in order to validate the selected turbulence model. Kumar et al.^54^, studied the turbulent flow in a tube-in-tube helical heat exchanger. Water was employed as both hot and cold fluids which were injected from opposite directions. The hot and cold fluids temperature was 323 K and 300 K, respectively. The Reynolds number for the hot fluid varied from 3100 to 5700, and 21,000 to 35,000 for the cold fluid. The Dean number for the hot fluid was 550–1000, and 3600–6000 for the cold fluid. The diameter of the inner tube (for hot fluid) and outer tube (for cold fluid) were 0.0254 m and 0.0508 m, respectively. The helical coil diameter and pitch were 0.762 m, and 0.100 m. Figure 3b shows the comparison of the experimental and present results in terms of various Nusselt numbers and Dean numbers for hot fluid at the inner tube. Three different turbulent models were performed and compared with experimental results. As shown in Fig. 3b, the results from the realizable k–ε turbulence model obtain a good agreement with experimental data. Therefore, this model was selected for this present study.

#### Numerical schemes

The numerical simulation in the present study is performed by utilizing the ANSYS Fluent 2020 R2. User-defined functions (UDFs) were written and applied as a source term of the energy equation in order to calculate the kinetic characteristics of the absorption process. The PRESTO scheme^55^ and PISO method^56^ are employed for the pressure–velocity coupling and pressure correction. The Green-Gauss cell-base is chosen for the variable’s gradients. The momentum and energy equations are solved by the second-order upwind method. In terms of under relaxation factors, 0.5, 0.7, 0.7 are set for pressure, velocity components and energy, respectively. The standard wall function was applied for the HTF in the turbulence model.

## Results and discussion

This section provides the results of numerical simulation of the heat transfer improvement inside the MH reactor by using a helical coil heat exchanger (HCHE) and semi-cylindrical coil heat exchanger (SCHE) for the hydrogen absorption process. The effect of the HTF pitch on the reactor bed temperature and absorption duration is analyzed. The critical operating parameters for the absorption process are investigated and presented in the sensitivity analysis section.

### Geometrical parameters

#### Effect of helical coil pitch

**Figure 4:**
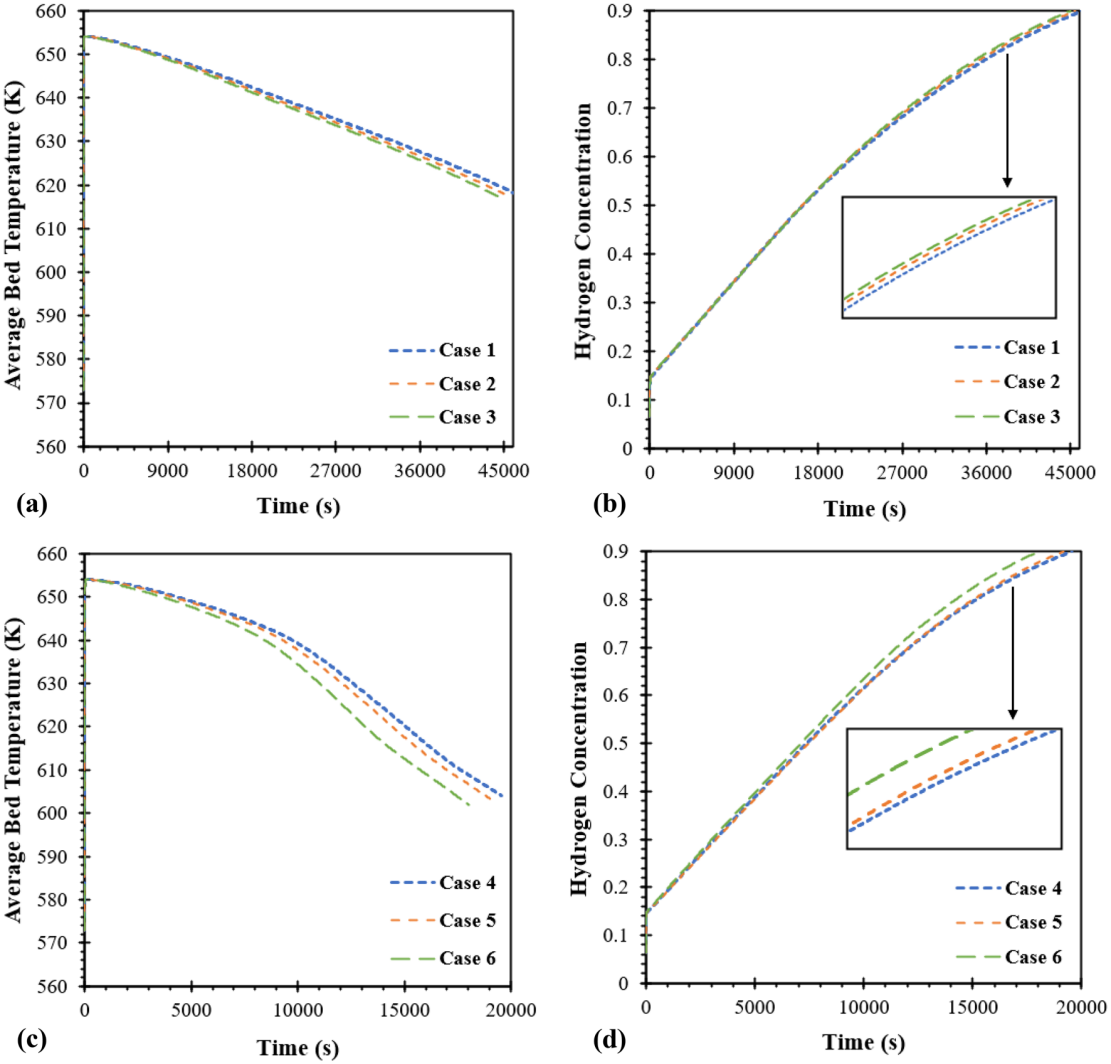
Effect of coil pitch on average bed temperature and hydrogen concentration. (**a**) Average bed temperature for helical coil pitch, (**b**) hydrogen concentration for helical coil pitch, (**c**) average bed temperature for semi-cylindrical coil pitch, and (**d**) hydrogen concentration for semi-cylindrical coil pitch.

Three heat exchanger configurations with different pitches were examined in order to study the effect of coil pitch on the heat transfer in the MH reactor. Three different pitches of 15 mm, 12.86 mm, and 10 mm are assigned as case 1, case 2, and case 3, respectively. It should be noted that the tube diameter is fixed as 6 mm under the initial temperature of 573 K and loading pressure of 1.8 MPa for all cases**.** Figure 4 presents the average bed temperature and hydrogen concentration of the MH bed during the hydrogen absorption process for case 1 to case 3. In general, the reaction between metal hydride and hydrogen is exothermic for the absorption process. Consequently, the bed temperature rapidly increases due to the initial moments when hydrogen is first injected into the reactor. The bed temperature is continually increasing until reaching the maximum value and gradually decreasing because the heat is removed by the HTF, which has a lower temperature and acts as a cooling fluid. As shown in Fig. 4a, the bed temperature rapidly increases and continually decreases due to the previous explanation. The hydrogen concentration for the absorption process is usually based on the bed temperature of the MH reactor. When the average bed temperature decreases to certain temperatures, the metal surface will absorb the hydrogen. This is because of the acceleration of physisorption, chemisorption, diffusion of hydrogen and its hydride formation in the reactor^36^. It can be seen in Fig. 4b, the rate of hydrogen absorption from case 3 is lower than other cases due to having a lower pitch value of the coil heat exchanger. This results in higher tube length in total and higher heat transfer area of HTF tube. The absorption time from case 1 is 46,276 s for the average hydrogen concentration as 90%. Comparing to the absorption duration from case 1, the absorption duration for case 2 and case 3 decreases 724 s and 1263 s, respectively. The temperature contours and hydrogen concentration contours at selected locations of the HCHE-MH bed are provided in the Supplementary section.

#### Effect of semi-cylindrical coil pitch

To improve the heat transfer performance of the MH reactor, two SCHEs are designed under a constant volume of MH (2000 cm^3^) and helical coil heat exchanger (100 cm^3^) from case 3. This section also considers the effect of coil pitch as 15 mm for case 4, 12.86 mm for case 5, and 10 mm for case 6. Figure 4c,d presents the average bed temperature and concentration for the hydrogen absorption process based on initial temperature at 573 K and loading pressure at 1.8 MPa. According to the average bed temperature from Fig. 4c, a lower coil pitch from case 6 significantly results in lower temperature compared to the other two cases. The lower bed temperature leads to higher hydrogen concentrations (see Fig. 4d) for case 6. The hydrogen absorption time for case 4 is 19,542 s which is over 2-times lower than using HCHE as case 1–3. In addition, the absorption time with lower pitch values from case 5 and case 6 also reduces 378 s and 1515 s compared to case 4. The temperature contours and hydrogen concentration contours at selected locations of the SCHE-MH bed are provided in the Supplementary section.

#### Performance comparisons between the MH reactors with helical coil heat exchanger and semi-cylindrical coil heat exchanger

**Figure 5:**
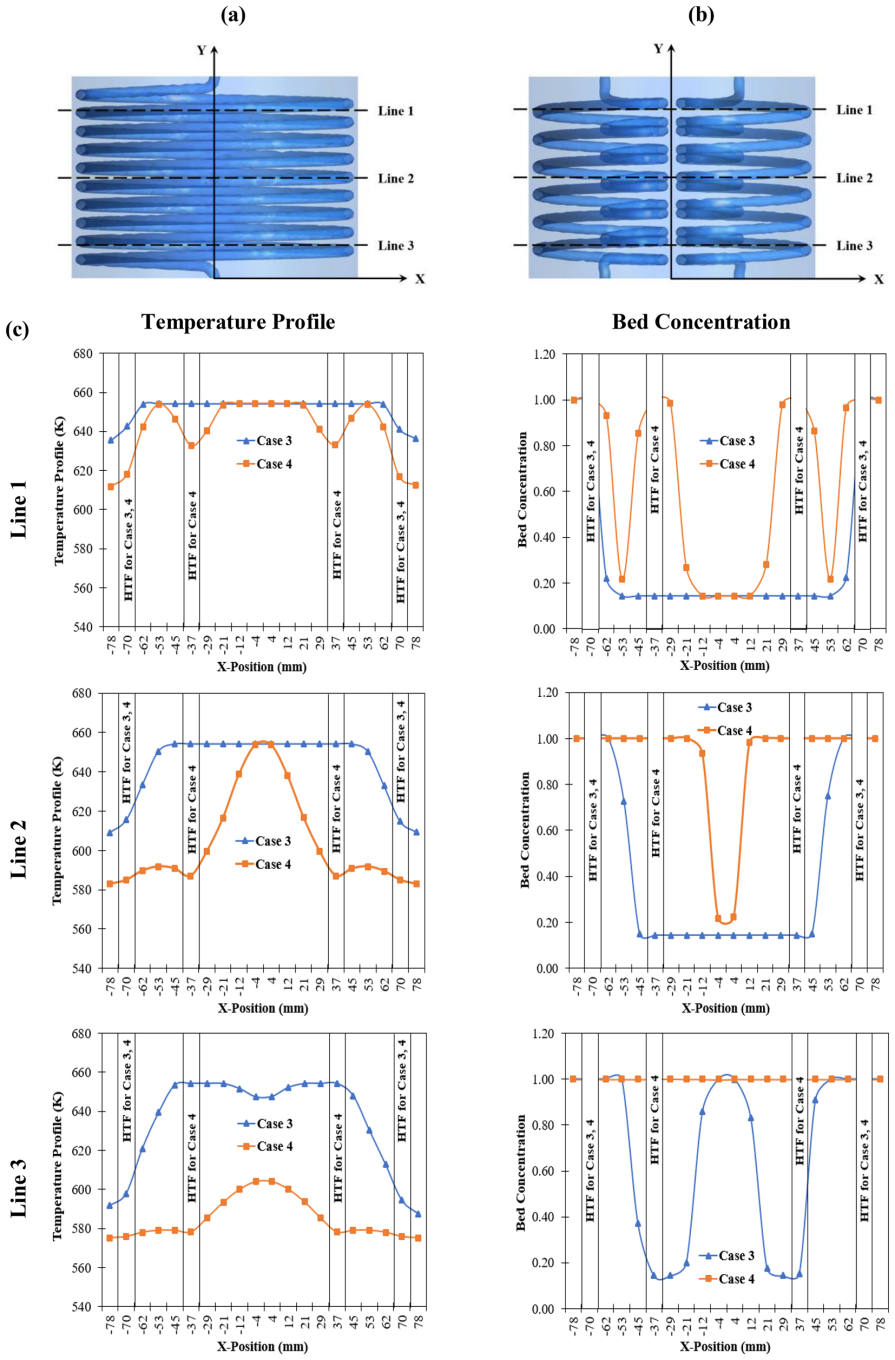
Temperature profiles and bed concentration at selected locations of the metal hydride reactor. (**a**) Selected locations for case 3, (**b**) selected locations for case 4, and (**c**) temperature profiles and bed concentration at selected locations after 20,000 s of hydrogen absorption process for case 3 and case 4.

To study the performance of two heat exchanger configurations, the temperature profiles at three selected locations are made and presented in this section. The MH reactor with HCHE from case 3 is selected to compare with the MH reactor incorporated SCHE from case 4 as having constant MH volume and tube volume. Operating conditions for this comparison are 573 K as an initial temperature and 1.8 MPa as loading pressure. Figure 5a,b presents all three selected locations for temperature profiles from case 3 and case 4, respectively. Figure 5c represents temperature profiles and bed concentration after 20,000 s of the hydrogen absorption process. According to Line 1 from Fig. 5c, the temperature around HTF from case 3 and case 4 reduces because of having convective heat transfer from the cooling fluid. This leads to a higher hydrogen concentration around this area. However, using two SCHEs results in higher bed concentration. A more rapid kinetic reaction was found around the HTF area for case 4. Furthermore, a maximum concentration of 100% was also found around this area. From Line 2, located at the middle part of the reactor, the temperature from case 4 is significantly lower than for case 3 for all locations except at the center of the reactor. This leads to the maximum amount of hydrogen concentration for case 4 excepted around the center of the reactor where it is far away from the HTF. However, the concentration for case 3 is insignificantly changed. The huge difference in temperature and bed concentration was observed at Line 3, which is near the HTF inlet. The bed temperature from case 4 significantly reduces, resulting in full hydrogen concentration at this area, while the concentration line from case 3 still fluctuates. This is due to the heat transfer acceleration from SCHEs. The details and discussion regarding the comparison of the average temperature of the MH bed and the HTF tube between case 3 and case 4 are provide in the Supplementary section.

**Figure 6:**
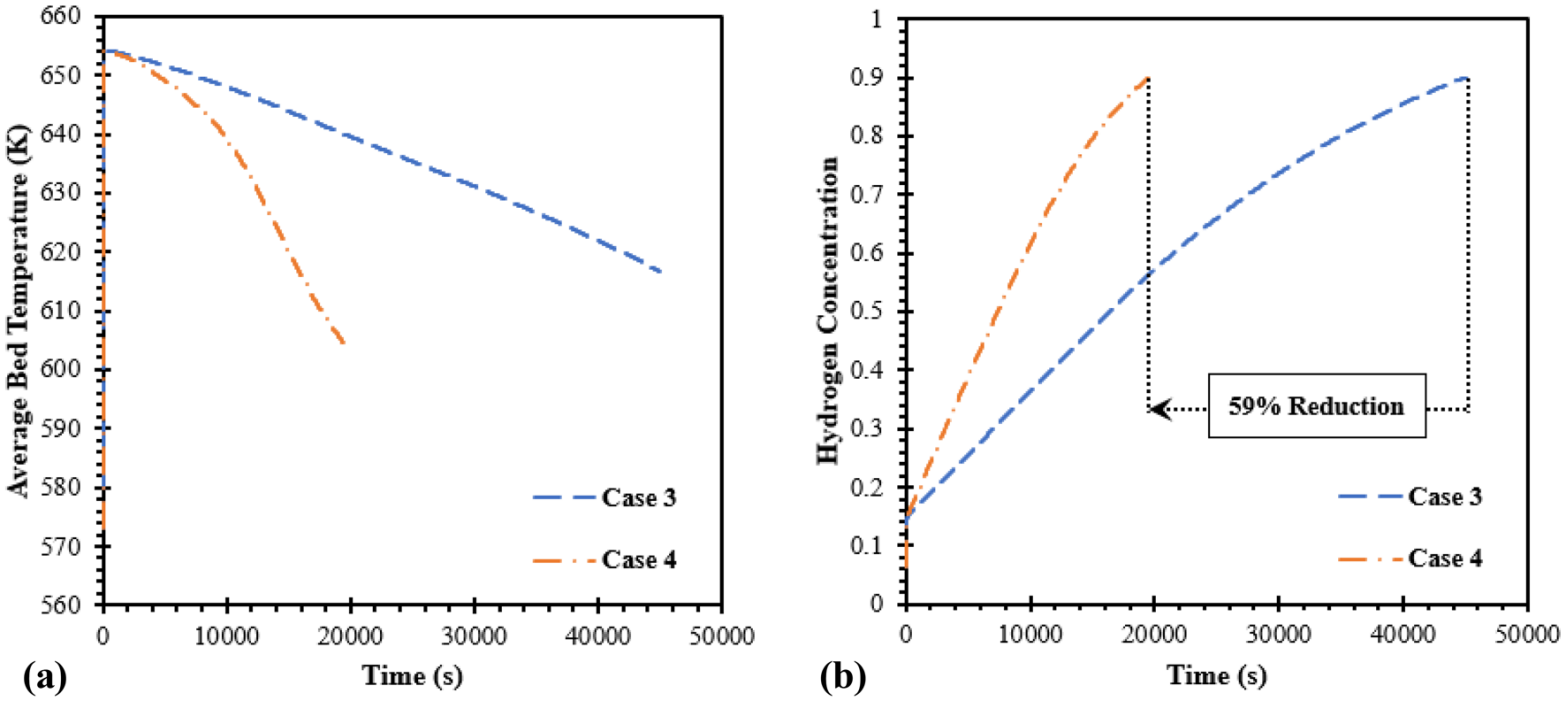
Comparison of average bed temperature and hydrogen concentrations between two heat exchanger configurations as case 3 and case 4.

**Figure 7:**
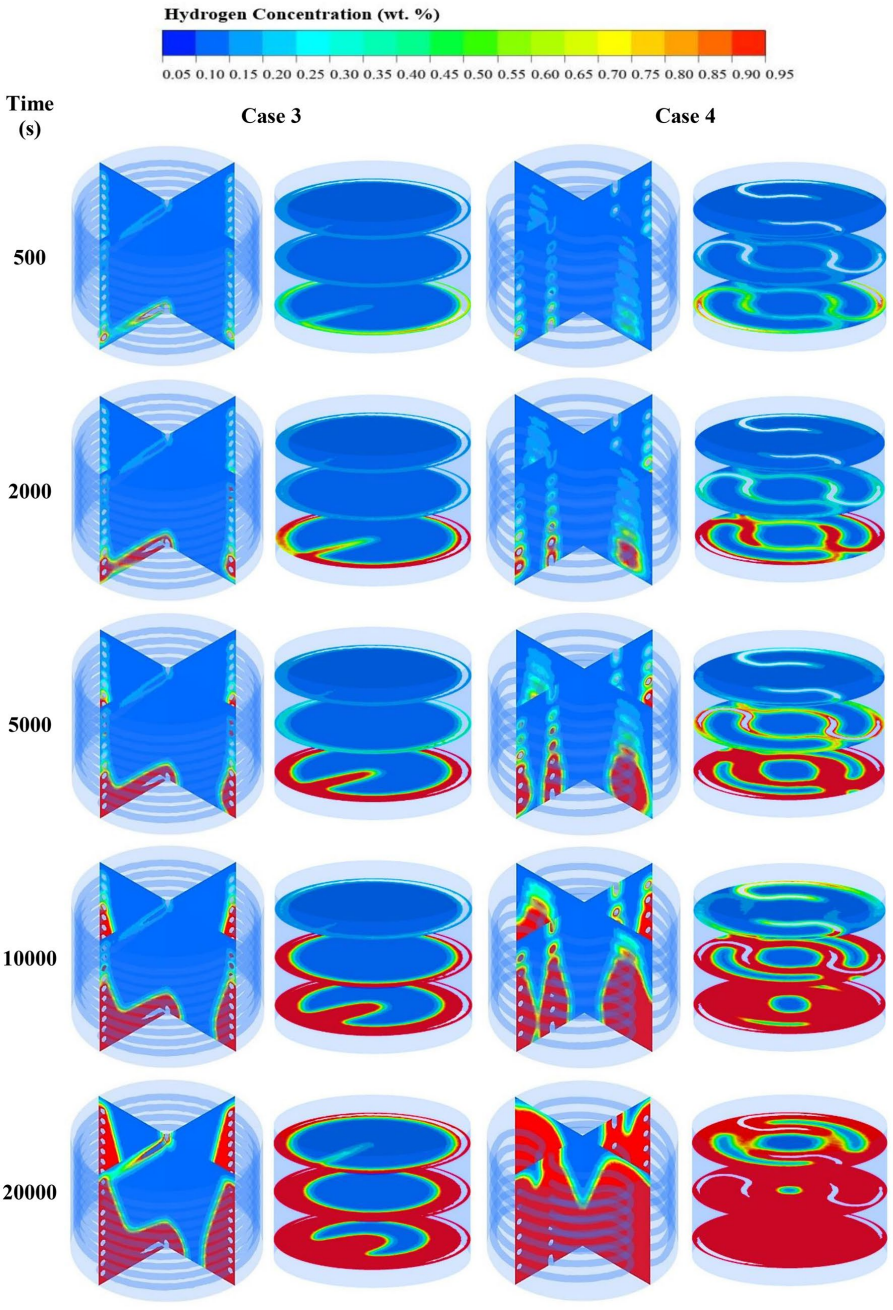
Comparison of hydrogen concentrations at 500 s, 2000s, 5000 s, 10,000 s, and 20,000 s after the start of the hydrogen absorption process between case 3 and case 4.

Figure 6 displays the comparison of average bed temperatures (see Fig. 6a) and hydrogen concentrations (see Fig. 6b) during the absorption process between HCHE and SCHE. From this figure, it is evident that the MH bed temperature significantly reduces because of an increasing of heat transfer area. Having more heat removal rate from the reactor leads to a faster hydrogen absorption rate. Although both heat exchanger configurations have a similar volume, the hydrogen absorption time based on SCHE as case 4 significantly reduces at 59% compared to using HCHE as case 3. For more analysis, the hydrogen concentrations from both heat exchanger configurations are displayed as contours in Fig. 7. This figure shows that the hydrogen starts to be absorbed in the bottom part around the HTF inlet for both cases. A higher concentration was found at HTF areas, while a lower concentration was observed at the center of the MH reactor due to being far away from the heat exchangers. At 10,000 s, the hydrogen concentration from case 4 is significantly higher than case 3. At 20,000 s, the average hydrogen concentration inside the reactor rises to 90% for case 4, while there is only 50% hydrogen for case 3. This can be explained by the reason that incorporating two SCHEs have a higher effective heat removal which leads to having lower temperature inside the MH bed. Thus, more equilibrium pressure declines inside the MH bed and then causes faster hydrogen absorption.

Table 5 summarizes the hydrogen absorption durations for all cases. Moreover, the hydrogen absorption time in percentage is also presented in this table. The percentage is calculated based on the absorption time from case 1. From this table, the absorption time from the MH reactor with HCHE is around 45,000 to 46,000 s, while the absorption time incorporating SCHE is around 18,000 to 19,000 s. When compared to case 1, the absorption time from case 2 and case 3 reduces only 1.6% and 2.7%, respectively. By employing SCHE instead of HCHE, the absorption time significantly reduces by 58 to 61% from case 4 to case 6. It is evident that incorporating SCHE inside the MH reactor significantly enhances the hydrogen absorption process and MH reactor performance. Although inserting the heat exchanger inside the MH reactor will reduce the storage capacity, this technique obtains a significant heat transfer improvement compared to other techniques. Moreover, the reduction of pitch values will increase the SCHE volume which leads to reducing the MH volume. In case 6, which has the highest SCHE volume, there is only a 5% reduction in the MH volume capacity compared to case 1, which has the lowest HCHE volume. Furthermore, during the absorption process, case 6 indicates faster and better performances with a reduction of 61% in absorption duration. Therefore, case 6 is selected to further investigation regarding sensitivity analysis. It should be noted that the long hydrogen absorption time is due to the storage capacity which contains the MH volume at around 2000 cm^3^.

**Table 5:** Summary of hydrogen absorption times for all six cases.

Case	Absorption time (s)	Absorption duration (%)
1	46,276	100
2	45,552	98.4
3	45,013	97.3
4	19,542	41.4
5	19,164	41.3
6	18,027	38.9

(The percentage of hydrogen absorption duration is calculated based on the absorption time from case 1).

### Sensitivity analysis of operation conditions

**Figure 8:**
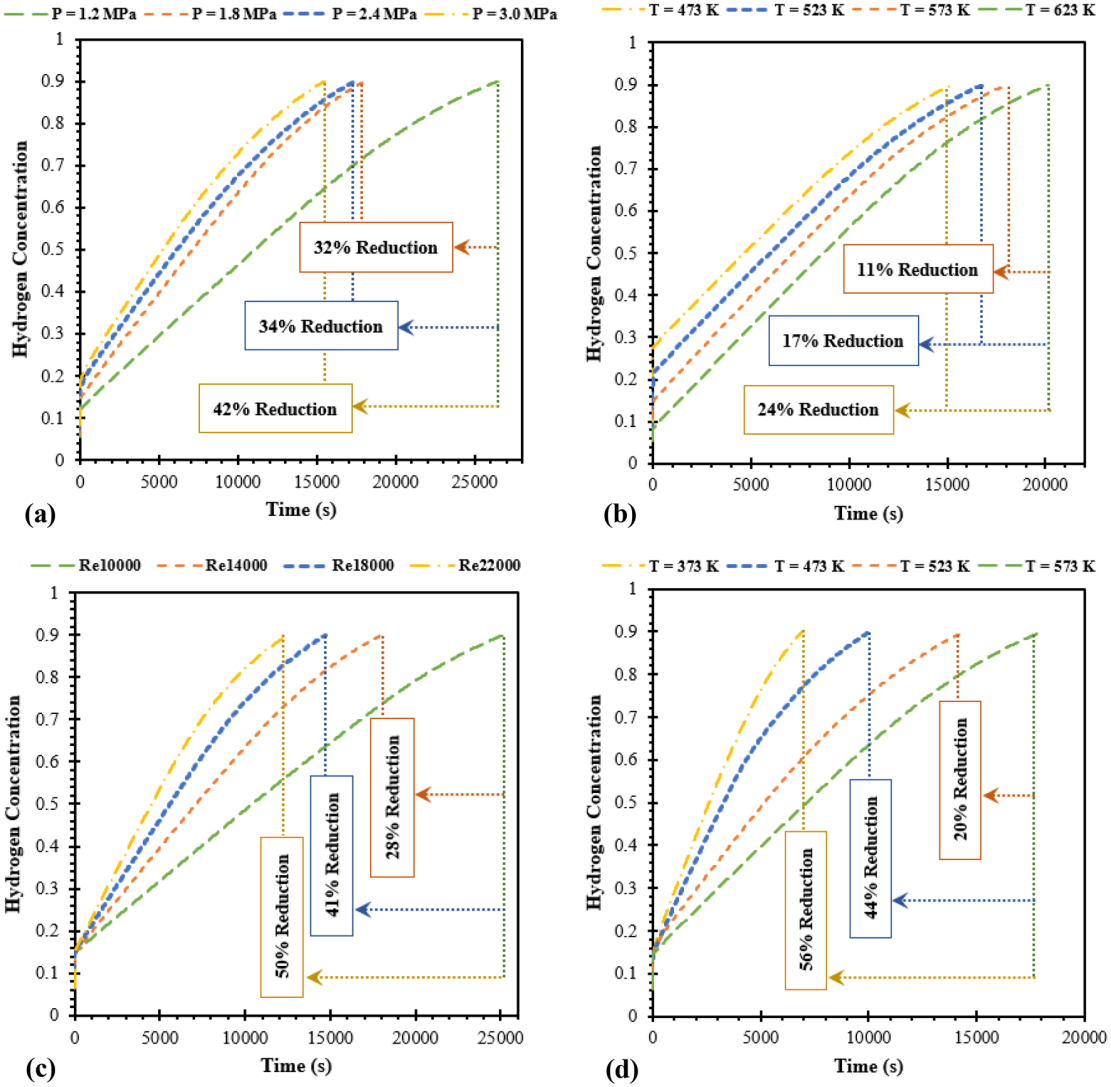
Hydrogen concentration diagram for various operating conditions in the use with the semi-cylindrical coil heat exchanger. (**a**) Loading pressures, (**b**) initial bed temperatures, (**c**) Reynolds numbers of heat transfer fluid, and (**d**) inlet temperatures of heat transfer fluid.

The operating parameters during the reaction process are essential factors that can be positively or negatively impact the performance of MH reactor in actual utilization. The sensitivity analysis is considered in this study to identify appropriate initial values of operating parameters for the MH reactor that is incorporated with SCHE, this section investigates four main operating parameters based on the best reactor configuration from case 6. The results from all operating conditions are presented in Fig. 8.

#### Effect of the loading pressure on the hydrogen absorption process

Four different loading pressures of 1.2 MPa, 1.8 MPa, 2.4 MPa, and 3.0 MPa were chosen based on the constant initial temperature at 573 K and HTF flow velocity at 14,000 of Reynolds number. Figure 8a reveals the effect of loading pressure and the SCHE on the hydrogen concentration with respect to time. The absorption time is reduced by an increment of loading pressure. Employing exerted hydrogen pressure with 1.2 MPa is the worst case for the hydrogen absorption process with the absorption duration over than 26,000 s for achieving 90% of hydrogen absorption. However, higher loading pressure results in the reduction of absorption times by 32 to 42% from 1.8 to 3.0 MPa. This is due to higher initial hydrogen pressure causing a larger difference between the equilibrium pressure and exerted pressure. Thus, this generates a greater driving force for hydrogen absorption kinetic^25^. At the initial moment, the hydrogen is rapidly absorbed because of the greater difference between the equilibrium pressure and exerted pressure^57^. With loading pressure at 3.0 MPa, 18% of the hydrogen is rapidly stored within the first 10 s. The hydrogen is stored at 90% of the reactor at the final stage with 15,460 s. However, the absorption time is significantly reduced by 32% from the loading pressure at 1.2 to 1.8 MPa. Other higher pressures have less effect on the improvement of the absorption time. Consequently, the loading pressure at 1.8 MPa is recommended for the MH-SCHE reactor. The hydrogen concentration contours for various loading pressures at 15,500 s are provided in the Supplementary section.

#### Effect of the initial temperature on the hydrogen absorption process

Selecting the appropriate initial temperature of the MH reactor is one of the main factors that influence the hydrogen sorption process, as it will affect the driving force of the hydride-producing reaction. To study the effect of SCHE on the initial temperature of the MH reactor, four different temperatures were chosen under constant loading pressure at 1.8 MPa and Reynolds number at 14,000 of HTF. Figure 8b presents the comparison of various initial temperatures, including 473 K, 523 K, 573 K, and 623 K. In fact, the Mg_2_Ni alloy will have effective performance for the hydrogen absorption process when the temperature is above 230 ℃ or 503 K^58^. However, the temperature will rapidly increase at the initial moment when hydrogen is injected. Thus, the MH bed temperature will be over 523 K. For this reason, hydride formation is then promoted due to the absorption rate enhancement^53^. From Fig. 8b, the hydrogen is absorbed faster when the initial temperature of the MH bed is reduced. When having a lower initial temperature, it leads to generating lower equilibrium pressure. The larger different pressures between equilibrium pressure and exerted pressure cause a faster hydrogen absorption process. By 473 K initial temperature, the hydrogen is rapidly absorbed to 27% within the first 18 s. Moreover, the absorption time from lower initial temperatures is also reduced from 11 to 24% compared to the initial temperature at 623 K. The absorption time with the lowest initial temperature at 473 K is 15,247 s which is similar to the best case of loading pressure. However, reducing initial reactor temperature results in lower hydrogen storage capacity. The initial temperature of MH reactor should not be less than 503 K^53^. Furthermore, the maximum hydrogen storage capacity of 3.6 wt% can be achieved by using the initial temperature of 573 K^53^. Focusing on the hydrogen storage capacity and the duration of the absorption, there is only a 6% time reduction by the temperature between 523 and 573 K. Therefore, the temperature at 573 K is recommended for the initial temperature of the MH-SCHE reactor. However, the effect of initial temperature on the absorption process is less significant compared to loading pressure. The hydrogen concentration contours for various initial temperatures at 15,500 s are provided in the Supplementary section.

#### Effect of the Reynolds number of the heat transfer fluid on the hydrogen absorption process

The flow velocity is one of the essential parameters for both hydrogeneration and dehydrogenation because of its ability that affect the turbulence and heat removal or heat supplying regarding hydriding and dehydriding processes^59^. A large flow velocity will generate a turbulent stage and cause faster fluid flow through the HTF tube. This reaction will result in faster heat transfer. Various inlet velocities of HTF are calculated based on the Reynolds number as 10,000, 14,000, 18,000, and 22,000. The initial temperature of MH bed is fixed at 573 K with the loading pressure at 1.8 MPa. The result from Fig. 8c proves that utilizing a higher Reynolds number incorporated with the SCHE leads to a faster absorption rate. With the increase of the Reynolds number from 10,000 to 22,000, the absorption time reduces approximately 28 to 50%. The absorption time from the Reynolds number at 22,000 is 12,505 s which is lower than the absorption time based on various initial temperatures and loading pressures. The hydrogen concentration contours for various Reynolds numbers of the HTF at 12,500 s are presented in the Supplementary section.

#### Effect of the initial temperature of the heat transfer fluid on the hydrogen absorption process

The effect of the SCHE on the initial HTF temperature is analyzed and displayed in Fig. 8d. Four initial temperatures of 373 K, 473 K, 523 K, and 573 K are chosen for this analysis under the initial MH temperature at 573 K and loading pressure of hydrogen at 1.8 MPa. Figure 8d indicates that the decrease in inlet HTF temperature leads to a shorter absorption time. Compared to the base case with inlet temperature at 573 K, the absorption time reduces around 20%, 44% and 56% for inlet temperature of 523 K, 473 K, and 373 K, respectively. At 6917 s with the initial temperature of the HTF at 373 K, there is a 90% of hydrogen concentration inside the reactor. This can be explained by the enhancement of convective heat transfer between the MH bed and the HTF. A lower HTF temperature will increase the heat removal rate and result in an improvement of the hydrogen absorption rate. Among all operating parameters, improving the MH-SCHE reactor’s performance by increasing the inlet temperature of the HTF is the most suitable method as the end of the absorption process is lower than 7000 s while the minimum absorption time from other methods is greater than 10,000 s. The hydrogen concentration contours for various initial temperature of the HTF at 7000 s are presented in the Supplementary section.

## Conclusion

The present study first introduces a novel semi-cylindrical coil heat exchanger embedded inside the metal hydride storage unit. The hydrogen absorption capacity of the proposed system is investigated under different heat exchanger configurations. The effect of operating parameters between the metal hydride bed and heat transfer fluid on the heat exchanged are examined, in order to find optimal conditions for the metal hydride storage with a novel heat exchanger. The key findings from this study are summarized as follows:

- Using a semi-cylindrical coil heat exchanger, heat transfer performance is improved as it has more uniform heat distribution in the magnesium bed reactor resulting in a better hydrogen absorption rate. Under the constant volume of the heat exchanger tube and metal hydride, the absorption reaction time is significantly reduced by 59% compared to a normal helical coil heat exchanger.
- Reducing the pitch size of coil heat exchangers positively affects the absorption duration because of having more heat transfer area. Among other pitch values, there is a 61% reduction of hydrogen absorption time when using semi-cylindrical coil heat exchangers with a pitch size of 10 mm. With this size, there is around a 5% reduction in the metal hydride volume capacity compared to the highest pitch size. Therefore, using a semi-cylindrical coil heat exchanger with 10 mm pitch size is recommended.
- Increasing the loading pressure of hydrogen injection leads to a lower hydrogen absorption time. The absorption duration significantly reduces, by 32%, with loading pressure at 1.8 MPa compared to 1.2 MPa. However, other higher values have less effect on the absorption duration. Therefore, the loading pressure at 1.8 MPa is recommended for the storage with a new heat exchanger.
- The lower initial temperature of the metal hydride bed results in a faster hydrogen absorption rate. However, to maintain the storage capacity with a Mg_2_Ni-based alloy, the initial temperature should not be less than 503 K. Considering the storage capacity and the absorption duration, the initial temperature at 573 K is recommended for the storage with a semi-cylindrical coil heat exchanger.
- The initial conditions of heat transfer fluid are the main parameters that significantly affect the improvement of storage performance with a novel heat exchanger. Higher Reynolds number of the heat transfer fluid positively influences hydrogen absorption duration because of having higher fluid flow velocity. Furthermore, a lower inlet temperature of heat transfer fluid also improves the convective heat transfer between the bed and the cooling fluid. By these two parameters, the absorption duration is significantly reduced by 50–56%.

The results from this study provide a heat transfer improvement regarding the absorption process of magnesium-based hydrogen energy storage under a novel heat exchanger configuration with optimized operating conditions. The comprehensive study on this proposed system could be beneficial for industrial applications. To improve the hydrogen absorption duration, the metal hydride storage with a novel semi-cylindrical coil heat exchanger will be further incorporated with other heat exchangers in the next study. Furthermore, the effect of using a novel heat exchanger on the hydrogen desorption process will be further considered.

## Supplementary Information

Supplementary Information.

## Data Availability

The datasets used and/or analysed during the current study available from the corresponding author on reasonable request.
