# Supplementary material for: Design optimization of a magnesium-based metal hydride hydrogen energy storage system
Source: Sci Rep. 2022 Aug 4;12:13436. doi: 10.1038/s41598-022-17120-3 (PMC9352733; doi:10.1038/s41598-022-17120-3)
Supplement: Supplementary file 1 — Supplementary Information. [file 41598_2022_17120_MOESM1_ESM.pdf]

Design Optimization of a Magnesium-based Metal Hydride Hydrogen Energy Storage System

Puchanee Larpruenrudee[1], Nick Bennett[1], YuanTong Gu[2], Robert Fitch[1], Mohammad S. Islam[1*]

[1]School of Mechanical and Mechatronic Engineering, University of Technology Sydney (UTS), 15 Broadway, Ultimo, NSW-2007, Australia

[2] School of Mechanical, Medical and Process Engineering, Faculty of Engineering, Queensland University of Technology, Brisbane-4000, Australia.

***Corresponding Author:** mohammadsaidul.islam@uts.edu.au

Supplementary Info File

System description

Metal hydride reactor with helical coil heat exchanger and semi-cylindrical coil heat exchanger

Supplementary Figure 1 presents a schematic diagram of MH reactors with a helical tube (**Supplementary Figure 1a**) and two semi-cylindrical tubes (**Supplementary Figure 1b**). From this supplementary figure., air as the HTF is injected from the bottom part into the porous MH reactor through a helical tube/two semi-cylindrical tubes, while hydrogen is injected from the upper surface of the reactor.

(a)

(b)

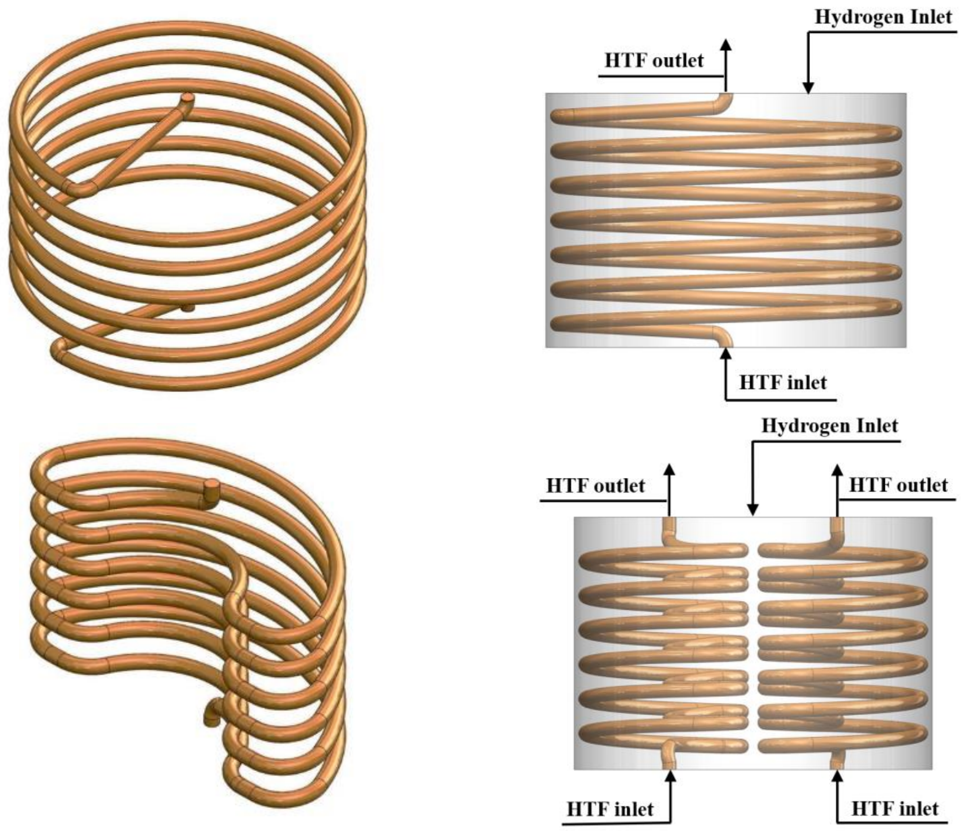

Supplementary Figure 1. Schematic diagram of MH reactors and characteristics of selected geometries for MH reactors. (a) With helical coil tub heat exchanger, and (b) With semi-cylindrical tube heat exchanger.

Mathematical Model

Governing equations

Heat transfer fluid

Equations used for heat transfer fluid are

$$\frac{\partial U_i}{\partial x_i} = 0 \,, \tag{1}$$

$$\rho U_i \frac{\partial U_i}{\partial x_i} = -\frac{\partial P}{\partial x_j} + \frac{\partial}{\partial x_i}\left[\mu\left(\frac{\partial U_i}{\partial x_j} + \frac{\partial U_j}{\partial x_i}\right) - \overline{\rho u_i' u_j'}\right] \quad , \tag{2}$$

$$\rho C_p U_i \frac{\partial T}{\partial x_i} = \frac{\partial}{\partial x_i}\left[\lambda \frac{\partial T}{\partial x_j} - \rho C_p \overline{u_i' T'}\right], \tag{3}$$

where U_i and T are the time-averaged velocity and temperature. The average Reynolds stresses and turbulent heat fluxes are defined as $\overline{\rho u_i' u_j'}$ and $\rho C_p \overline{u_i' T'}$, respectively.

The kinetic energy equation is expressed as:

$$\frac{\partial}{\partial t}(\rho k_t) + \frac{\partial}{\partial x_j}(\rho k_t u_j) = \frac{\partial}{\partial x_j}\left[\left(\mu + \frac{\mu_t}{\sigma_{k_t}}\right)\frac{\partial k_t}{\partial x_j}\right] + G_{k_t} + G_b - \rho\varepsilon_t - Y_M + S_{k_t} \tag{4}$$

The dissipation rate of the turbulent kinetic energy equation is defined as:

$$\frac{\partial}{\partial t}(\rho\varepsilon_t) + \frac{\partial}{\partial x_j}(\rho\varepsilon_t u_j) = \frac{\partial}{\partial x_j}\left[\left(\mu + \frac{\mu_t}{\sigma_{\varepsilon_t}}\right)\frac{\partial \varepsilon_t}{\partial x_j}\right] + \rho C_1 S\varepsilon_t - \rho C_2 \frac{\varepsilon_t^2}{k_t + \sqrt{v\varepsilon_t}} + C_{1,\varepsilon_t}\frac{\varepsilon_t}{k_t} C_{3,\varepsilon_t} G_b + S_{\varepsilon_t} \tag{5}$$

where the constant coefficients define as $C_1 = 1.47$, $C_2 = 1.92$, $\sigma_{k_t} = 1.0$, and $\sigma_{\varepsilon_t} = 1.3$. G_{k_t} and G_b are the turbulence kinetic energy production induced by mean velocity gradients and buoyancy, respectively. The contribution of the fluctuating dilatation in the incompressible turbulence to the overall dissipation rate is expressed as Y_M. The turbulent viscosity is defined as $\mu_t = \rho C_\mu \frac{{k_t}^2}{\varepsilon_t}$. The turbulent intensity is calculated based on the average Reynolds number at inlets by $I = 0.16\left(Re_{D_h}\right)^{-0.125}$.

Grid independency

The average bed temperature for the hydrogen absorption process for various mesh sizes are presented in **Supplementary Figure 2a, b** for case 1 and case 4, respectively. The average bed temperature of the absorption process after the element numbers 428891 (**Supplementary Figure 2a** for case 1) and 430599 (**Supplementary Figure 2b** for case 4) do not vary. Hence, these grid sizes are selected for further computational calculations. **Supplementary Figure 3** presents the successively refined grids for case 1 and case 4.

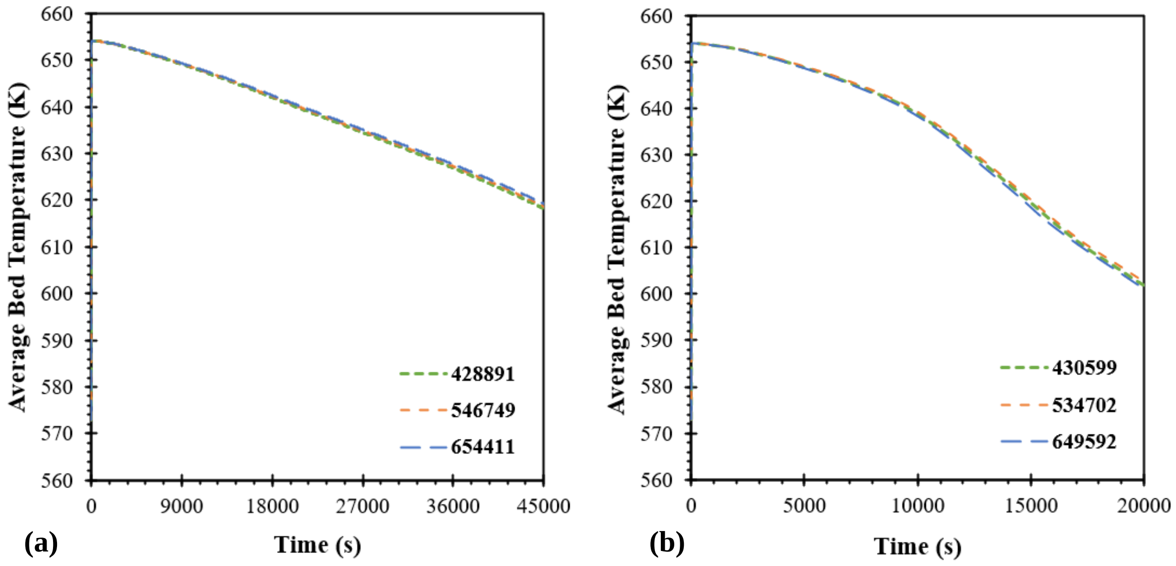

Supplementary Figure 2. Average bed temperature for the hydrogen absorption process in the MH reactor under various grid numbers. (a) average bed temperature for case 1, and (b) average bed temperature for case 4.

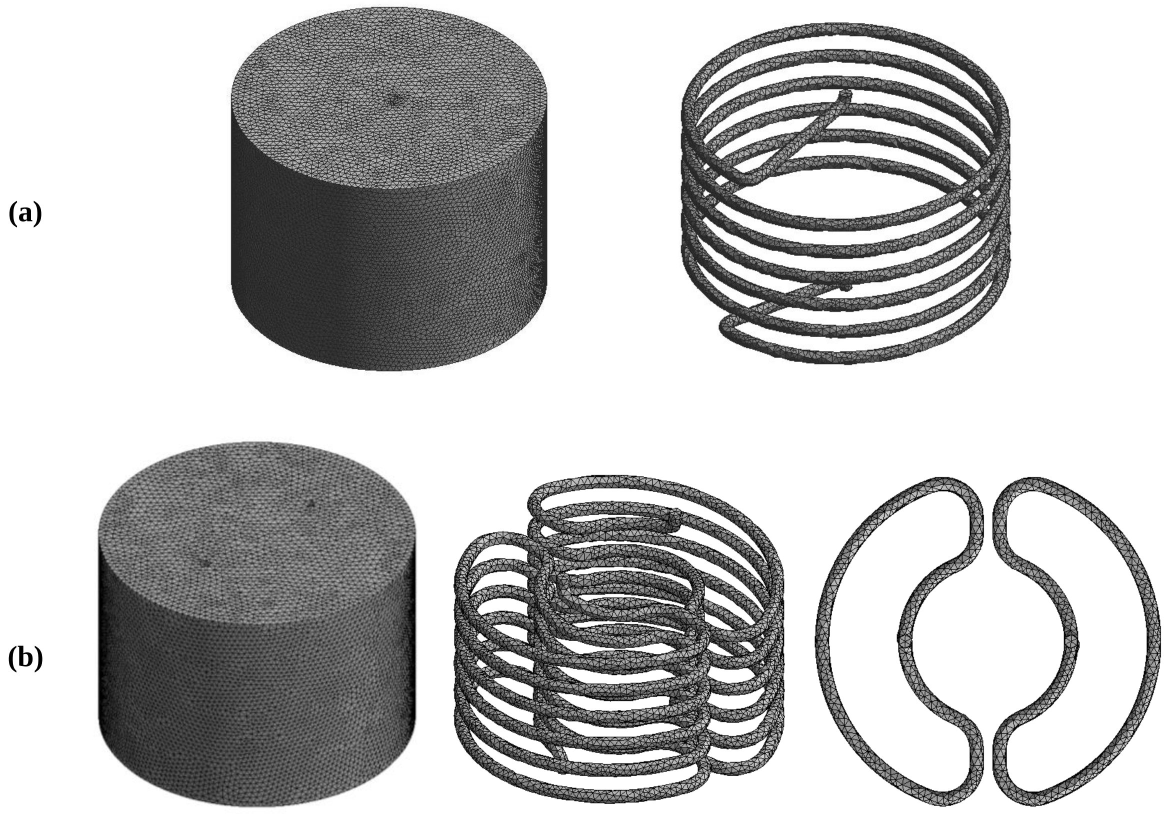

Supplementary Figure 3. Computational meshing of MH reactor and HTF tube. (a) case 1, and (b) case 4.

Results and discussion

Geometrical parameters

Effect of helical coil pitch

Supplementary Figure 4 shows the temperature contours at three selected locations of the MH bed for 500 s, 25000 s, and 45000 s. From this figure, the lower bed temperature was found at the bottom plane for all cases where it is near the HTF inlet. Nearly the HTF wall, the temperature significantly reduces while higher temperature locates at the center where it has no contact area with the HTF. For comparison, reducing pitch values results in greater heat transfer improvement, especially for case 3. **Supplementary Figure 5** demonstrates the hydrogen concentration at selected times for three pitch sizes. At 500 s, from **Supplementary Figure 5**, it can be seen that the hydrogen begins to get absorbed at the bottom part where it is around the HTF inlet. Similarly, higher hydrogen concentration was found around the HTF area at 25000 s and 45000 s. This is because of the conductive heat transfer mechanism between the HTF and MH reactor. Therefore, the center area of the reactor has less hydrogen concentration due to having less conductive heat transfer from the HTF.

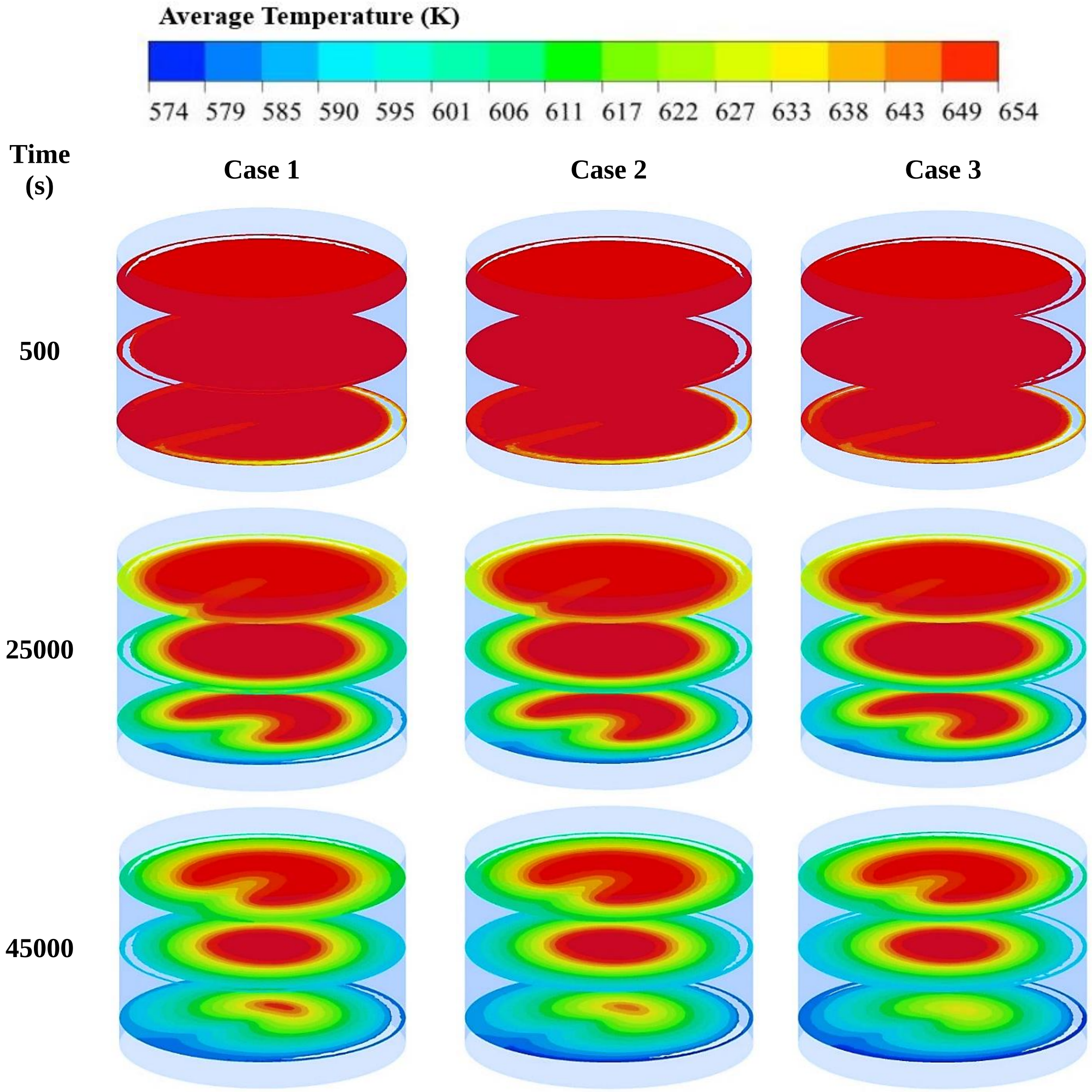

Supplementary Figure 4. Distribution of bed temperature at 500 s, 25000 s, and 45000 s after the start of the hydrogen absorption process in the MH reactor.

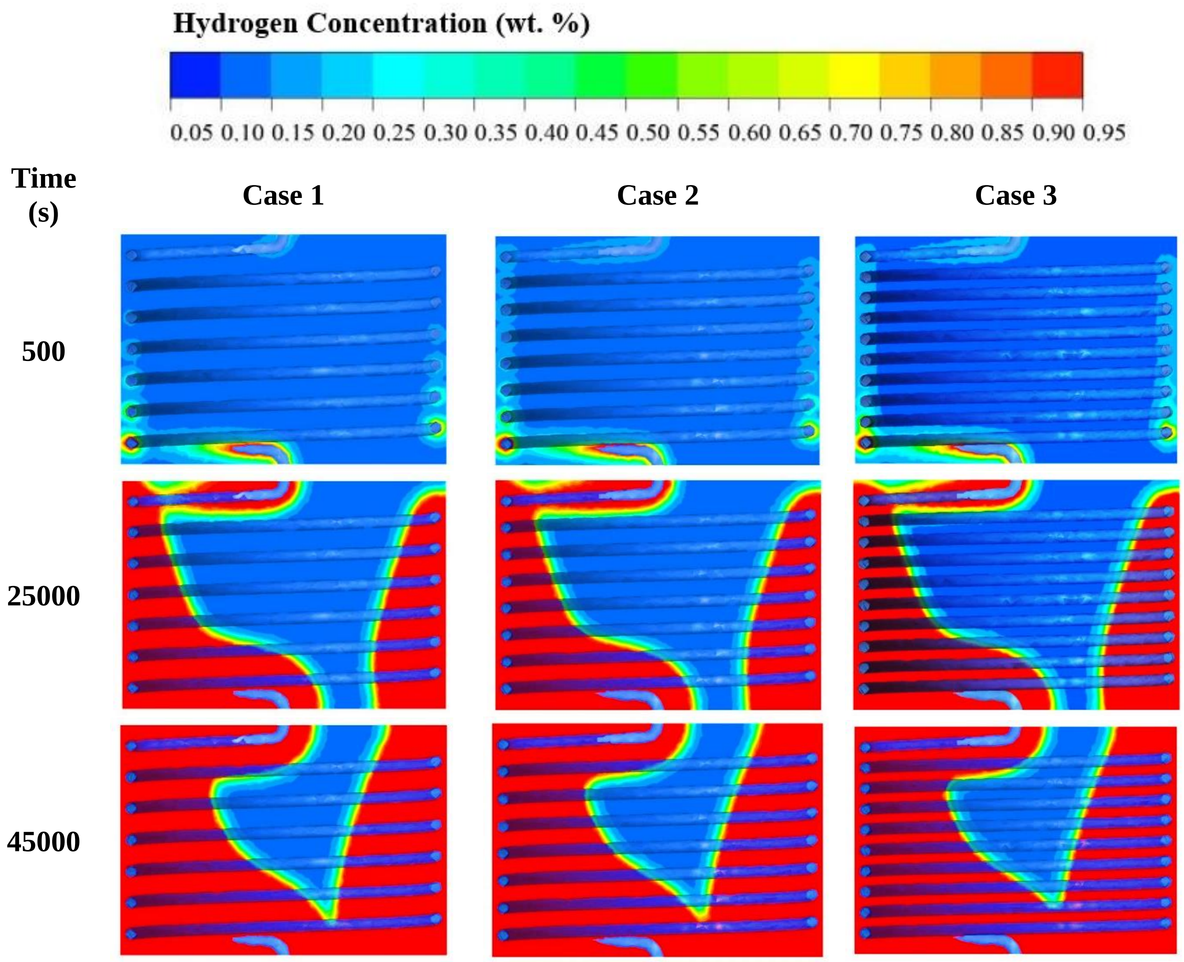

Supplementary Figure 5. Hydrogen concentration in the MH bed after 500 s, 25000 s, and 45000 s of the hydrogen absorption process in the MH reactor for different helical coil pitches.

Effect of semi-cylindrical pitch

Supplementary Figure 6 and **Supplementary Figure 7** demonstrate the average bed temperature and hydrogen concentration, respectively. The selected times as 500 s, 9000 s, and 18000 s for both bed temperature and concentration are provided for all three cases. From these two figures, a lower bed temperature and higher hydrogen concentration were found at the bottom section for all cases due to better conductive heat transfer. A higher temperature is also located at the center of MH reactor and the top, close to hydrogen injection. This results in lower hydrogen absorption in these areas. Similar to HCHE, higher hydrogen concentration was observed around the HTF area especially at the bottom reactor, where it has an HTF inlet.

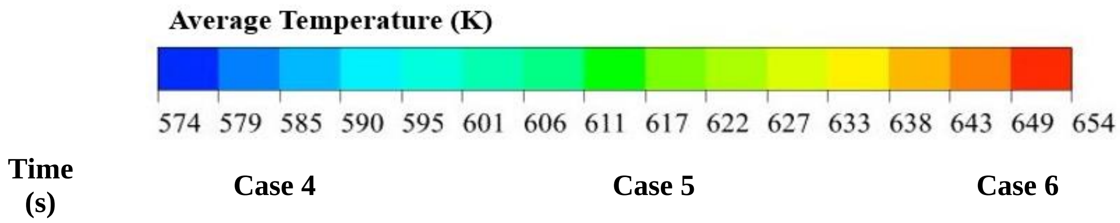

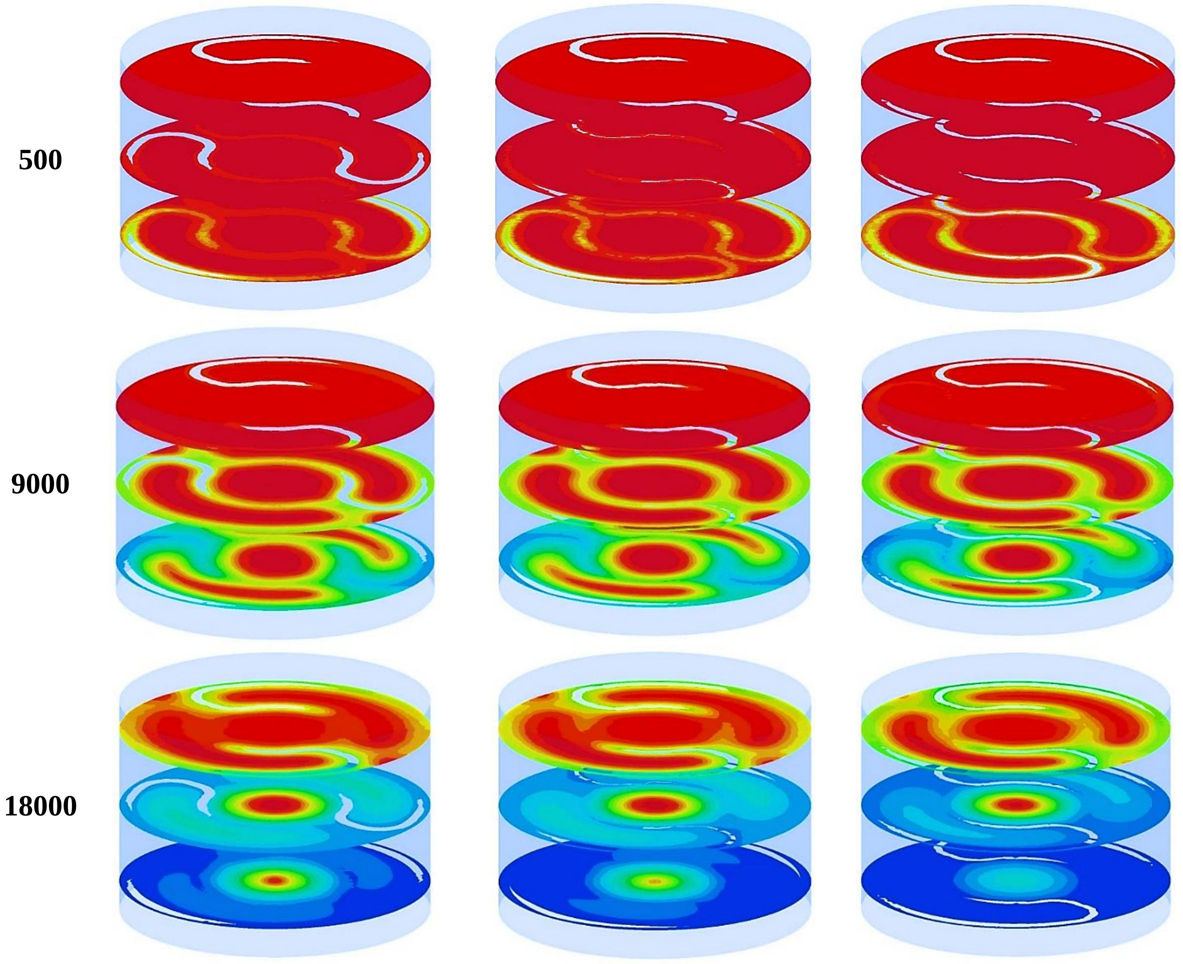

Supplementary Figure 6. Distribution of bed temperature at 500 s, 9000 s, and 18000 s after the start of the hydrogen absorption process in the MH reactor.

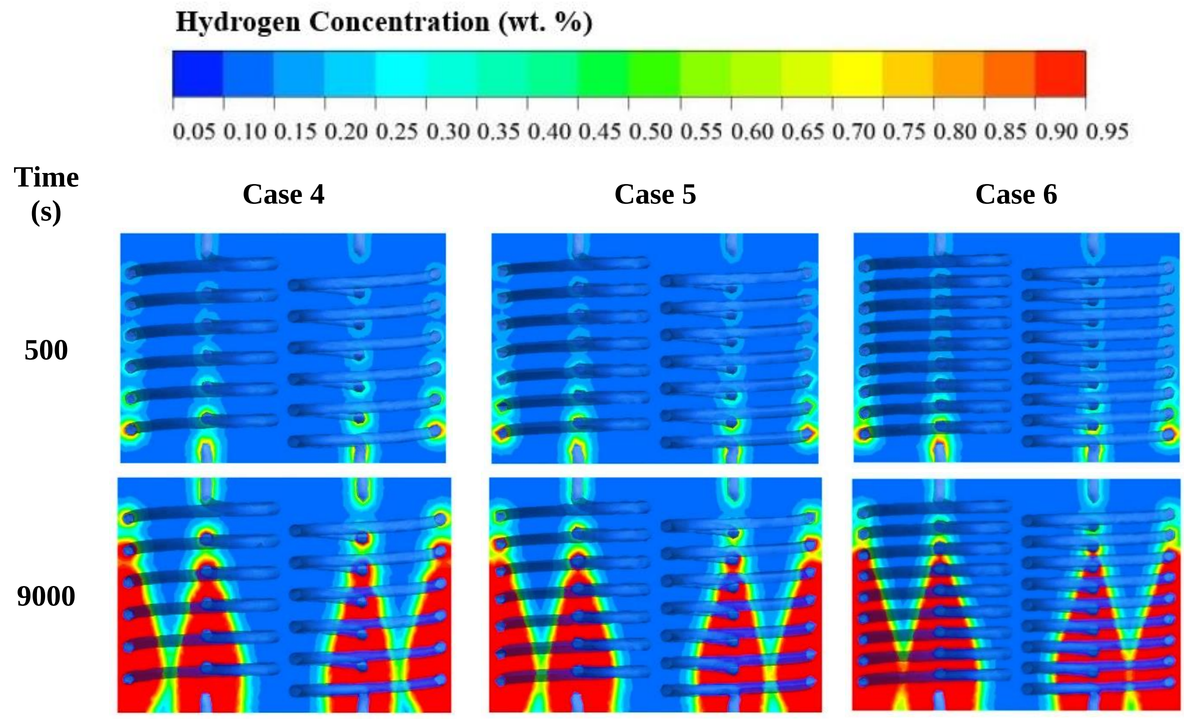

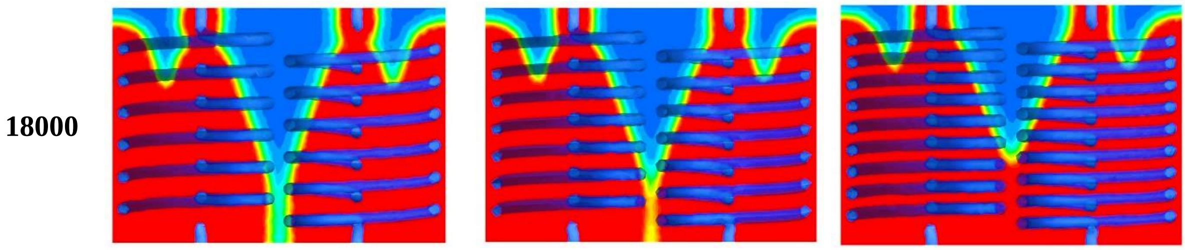

Supplementary Figure 7. Hydrogen concentration in the MH bed after 500 s, 9000 s, and 18000 s of the hydrogen absorption process in the MH reactor for different semi-cylindrical pitches.

Performance comparisons between the MH reactors with helical coil heat exchanger and semi-cylindrical coil heat exchanger

The comparison of the average temperature of the MH bed and the HTF tube between case 3 and case 4 is presented in **Supplementary Figure 8** in order to provide a better understanding of the relationship between the HTF and the MH bed in a systematic way. Various selected times after the start of the absorption process are included. For the HTF tube, the air as a cooling fluid is injected from the bottom and then flows towards the tube to the outlet at the top of the MH reactor. At the beginning, the average HTF around the upper part is higher than the lower part due to exposure to the reaction heat generated during the hydrogen absorption process from the top surface of the MH reactor. This results in higher average bed temperature of the MH reactor. Regarding the exothermic behavior of hydrogen absorption, the initial temperature of the reactor rapidly increases and is significantly higher than the HTF temperature. This will generate a high heat transfer rate between the HTF and MH bed. The average HTF temperature then continually decreases, which is related to absorption times. Therefore, conductive heat transfers between the MH bed and the HTF is occurring. A larger difference in temperature rates between the MH reactor and the HTF causes a higher heat transfer rate. At 10000 s and 20000 s, the bed temperature at the bottom and middle sections are reduced and close to the HTF average temperature. However, this is only for case 4. The average bed temperature inside the MH reactor with HCHE from case 3 is still high, even the bottom part of the reactor. The bed temperature difference between center and outer areas from case 3 is significantly higher than case 4. The existence of temperature gradients between these areas negatively affects mass transfer mechanisms and the overall MH reactor performance. Furthermore, the difference in heat transfer performance between these two configurations is mainly because of having less space between the HTF and the MH bed for SCHE that, leads to having more uniform temperature distribution as well as lower thermal resistance inside the reactor.

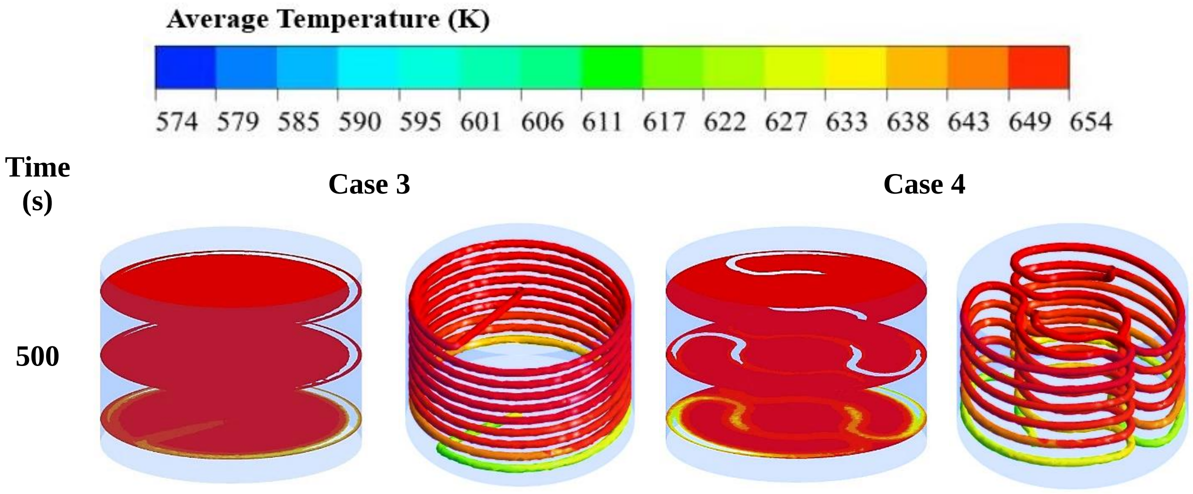

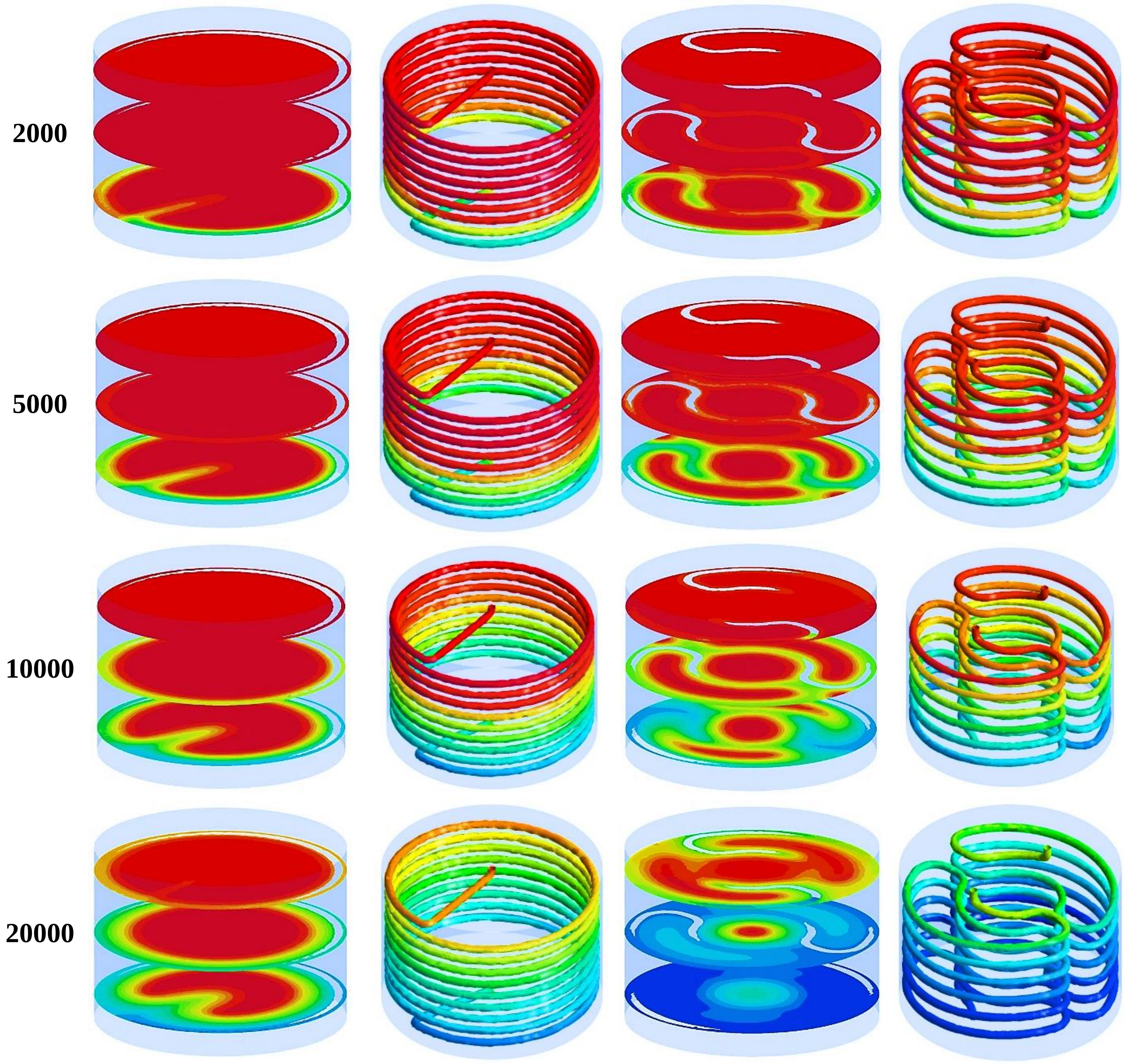

Supplementary Figure 8. Average temperature of MH bed and HTF tube at 500 s, 2000 s, 5000 s, 10000 s, and 20000 s after the start of the hydrogen absorption process in the MH reactor for case 3 and case 4.

Sensitivity analysis of operating conditions

Effect of the loading pressure on the hydrogen absorption process

Supplementary Figure 9 illustrates the hydrogen concentration at 15500 s for all loading pressures. It can be seen that there is a significantly different concentration between 1.2 MPa and 3.0 MPa.

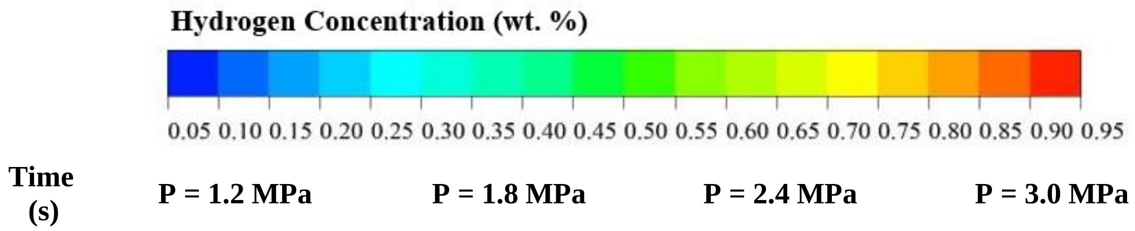

15500

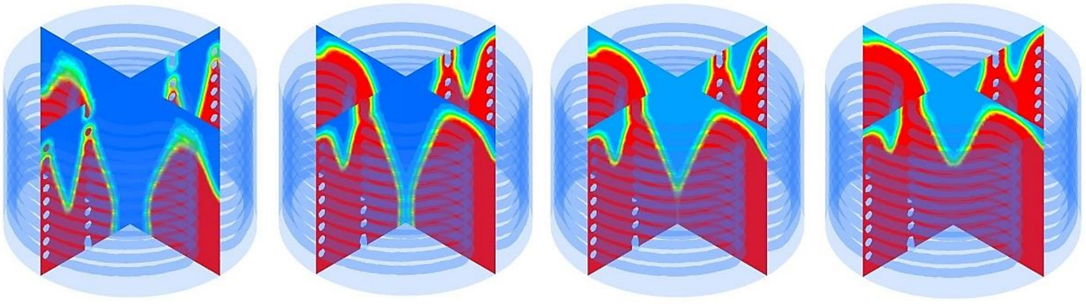

Supplementary Figure 9. Hydrogen concentration at 15500 s after start of the hydrogen absorption process for various loading pressures.

Effect of the initial temperature on the hydrogen absorption process

Supplementary Figure 10 illustrates the hydrogen concentration contours at 15500 s. It can be seen that the decreasing an initial temperature of MH bed also influences hydrogen concentration.

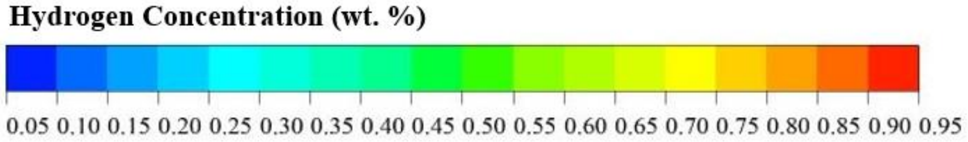

Time (s)	T = 473 K	T = 523 K	T = 573 K	T = 623 K

15500

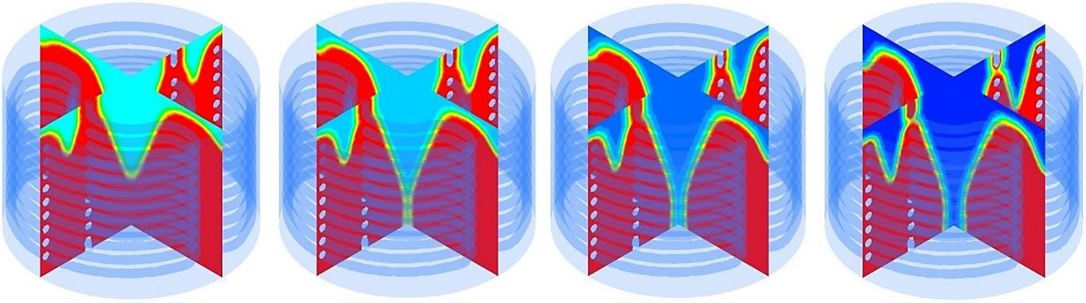

Supplementary Figure 10. Hydrogen concentration at 15500 s after start of the hydrogen absorption process for various initial bed temperature.

Effect of the Reynolds Number of the heat transfer fluid on the hydrogen absorption process

Supplementary Figure 11 presents the hydrogen concentration at 12500 s for all Reynolds numbers. Similar to other operating parameters, increasing the Reynolds number results in faster hydrogen absorption, especially for the case of Reynolds number as 22000 compared to 10000.

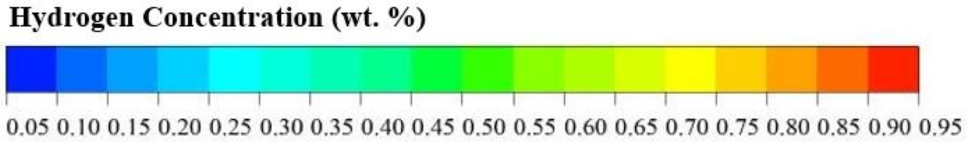

Time (s)	Re = 10000	Re = 14000	Re = 18000	Re = 22000

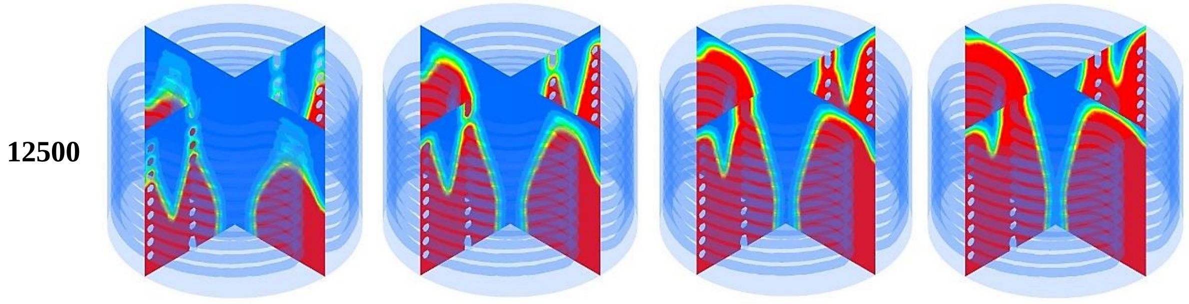

Supplementary Figure 11. Hydrogen concentration at 12500 s after start of the hydrogen absorption process for various Reynolds numbers of heat transfer fluid.

Effect of the initial temperature of the heat transfer fluid on the hydrogen absorption process

Supplementary Figure 12 illustrates the hydrogen concentration at 7000 s for all inlet HTF temperatures. It can be seen that over 90% of hydrogen is stored in the reactor with the inlet temperature at 373 K, whereas there is around 50% hydrogen stored in the reactor from 573 K inlet temperature.

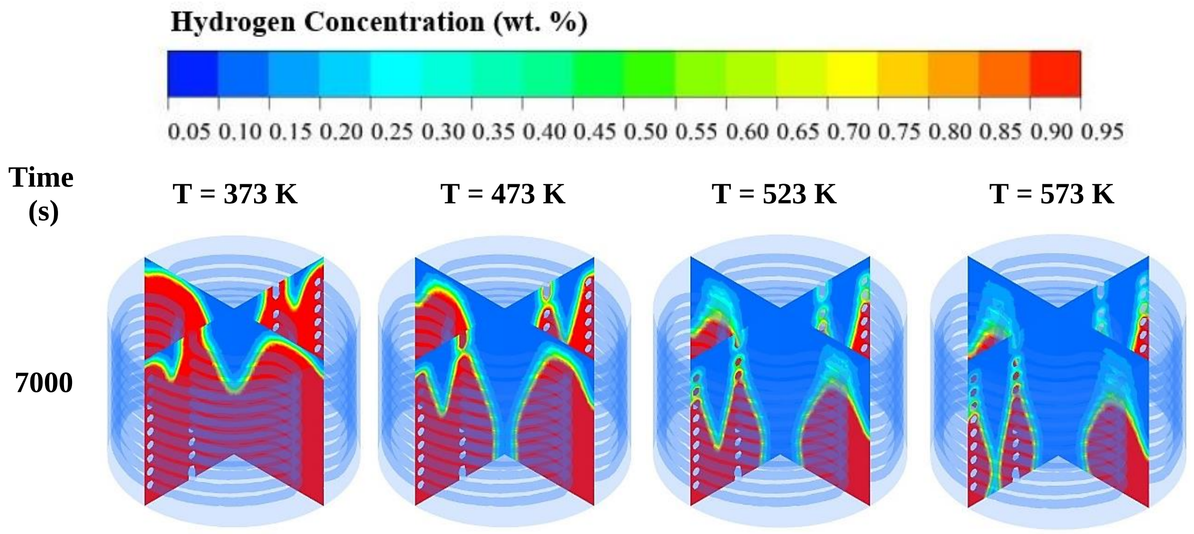

Supplementary Figure 12. Hydrogen concentration at 7000 s after start of the hydrogen absorption process for various inlet temperatures of heat transfer fluid.

Nomenclature

C_a	Reaction rate constant for absorption, s^{-1}
C_p	specific heat, J kg^{-1} K^{-1}
D_i	Inner diameter of coil tube, mm
D_o	Outer diameter of reactor, mm
D_p	Coil pitch length, mm
D_t	Diameter of tube, mm
E_a	Activation energy for absorption, J mol^{-1}
G	Gap between two semi-cylindrical coil heat exchanger, mm
G_{k_t}	Generation of turbulent kinetic energy due to mean velocity gradients
G_b	Generation of turbulent kinetic energy due to mean velocity gradients buoyancy
I	Turbulent intensity
K	Permeability, m^2
k_t	Turbulent kinetic energy
L_i	Coil length, mm
L_o	Reactor length, mm
M	Molecular weight, g mol^{-1}
$\vec{n}$	Normal vector
P	Pressure, MPa
P_0	Hydrogen exerting pressure, MPa
R	Universal gas constant, J K^{-1} mol^{-1}
R_i	Inner radius of semi-cylindrical coil heat exchanger, mm
R_o	Outer radius of semi-cylindrical coil heat exchanger, mm
Re	Reynolds number
S_{k_t}	Turbulent kinetic energy source term, J
S_{ε_t}	Turbulent dissipation source term, J
T	Temperature, K
T'	Fluctuating temperature, K
t	time, s
U	Time-averaged velocity, m s^{-1}
u_i'	Fluctuating velocity, m s^{-1}
X	Absorbed hydrogen amount, w%
x_f	Maximum absorbed hydrogen amount
Y_M	Contribution of the fluctuating dilatation in compressible turbulence to the overall dissipation rate
wt	Maximum mass content of hydrogen in the metal, w%
ΔH	Reaction heat, J mol^{-1}
ΔS	Reaction entropy, J mol^{-1} K^{-1}

Greek

ε	Porosity
ε_t	Turbulent dissipation rate
σ_{ε_t}	Turbulent Prandtl number for ε_t
σ_{k_t}	Turbulent Prandtl number for k_t
λ	Thermal conductivity, W m^{-1} K^{-1}
$\lambda_{e,MH}$	Effective thermal conductivity of metal hydride, W m^{-1} K^{-1}
μ	Dynamic viscosity, Pa s
v	Kinematic viscosity, m^2 s^{-1}
ρ	Density, kg m^{-3}
ρ_{MH}	Hydride density, kg m^{-3}
$\rho_{ss,MH}$	Density of saturated metal hydride, kg m^{-3}

Subscript

0	Initial or exerting condition
a	Ambient or absorption
e	Effective
eq	Equilibrium
f	Fluid
H_2	Hydrogen
i	Inner or Cartesian coordinate
J	Cartesian coordinate
in	Inlet
M	Metal
O	Outer
ref	Reference
s	Static

Abbreviation

MH	metal hydride
HTF	heat transfer fluid
HCHE	helical coil heat exchanger
SCHE	semi-cylindrical coil heat exchanger
